# White and fissured: the *Acarospora strigata* group (*Acarosporales*, *Acarosporaceae*) in North America

**DOI:** 10.3897/mycokeys.142.208759

**Published:** 2026-10-19

**Authors:** Kerry Knudsen, Jana Kocourková, Eva Kondrysová, Steve Leavitt, Jason Hollinger, Natassja Noell, Jiří Malíček, Tim Wheeler

**Affiliations:** 1 Department of Ecology, Faculty of Environmental Sciences, Czech University of Life Sciences Prague, Kamýcká 129, Praha - Suchdol, 165 00, Czech Republic Department of Ecology, Faculty of Environmental Sciences, Czech University of Life Sciences Prague Praha Czech Republic; 2 Department of Biology & M.L. Bean Life Science Museum, Brigham Young University, Provo, UT 84602, US Department of Biology & M.L. Bean Life Science Museum, Brigham Young University Provo United States of America; 3 Herbarium, Department of Biology, Western Carolina University, Cullowhee, NC 28723, USA Herbarium, Department of Biology, Western Carolina University Cullowhee United States of America; 4 Institute of Botany, The Czech Academy of Sciences, Zámek 1, CZK 252 43 Průhonice, Czech Republic Institute of Botany, The Czech Academy of Sciences Průhonice Czech Republic; 5 Division of Biological Sciences, University of Montana, Missoula, Montana, USA Division of Biological Sciences, University of Montana Missoula United States of America

**Keywords:** Asia, China, desert lichens, epitype, lichenicolous lichens, nomenclature, rare lichens

## Abstract

*Acarospora peltata* was synonymized by A. H. Magnusson with the South American species *A. strigata*. We do not recognize the synonymy. *Acarospora peltata* is the common North American species. *Acarospora strigata* is relatively rare in South America. *Acarospora peltastica* is recognized as a rare southern California species with globose ascospores (3.5–)4–5 μm in diameter. We describe the new species *A. lactea* from Montana, Nevada and Utah. We revise and publish new descriptions of *A. coloradiana*, *A. nevadensis*, and *A. utahensis*. A key is published for the six members of the *A. strigata* group in North America.

## Introduction

The morphologically defined *Acarospora strigata* group occurs in Asia, north Africa, Europe and South and North America (Magnusson 1929, 1940, 1944,1952, 1954, 1956; Knudsen 2007; Knudsen et al. 2015; Nurtai et al. 2017; Leavitt et al. 2018). They share a common phenotype especially adapted to arid and desert habitats (see images of species throughout paper). The areoles or squamules are white, with pruina in a polysaccharide epicortex 10–110 µm thick (Magnusson 1929; Knudsen 2007). Due to the well-known albedo effect, the white surface reflects sunlight and, thus, absorbs less heat, but the amount of heat radiated has never been measured in these species. We presume that the epicortex preserves the algal layer and conserves moisture, but that has not been studied either. Thalli have fissures in various patterns from radial to crosshatched. The fissures deepen becoming abscission fissures. The species commonly replicate by division forming a colony. The hypothallus below a colony of areoles or squamules can become visible as a common mycelial base as seen in Fig. 3. In our recent key of southwestern *Acarosporaceae*, the *Acarospora strigata* group is determined from all other species of *Acarosporaceae*, based on the pruinose, fissured thallus (Knudsen et al. 2023a).

There are 13 described species of this group worldwide. The first species described from the group was *Acarospora strigata*, which occurs in South America (Nylander 1855). Four species occur in Asia: *A. bicolor* from Siberia, *A. interrupta* from Mount Sinia in Egypt to China, *A. iqbalii* from Pakistan and China and *A. tianshanica* from China (Magnusson 1929; Nurtai et al. 2017; Iqbal et al. 2025; Liang et al. 2026). *Acarospora caeruleoalba* occurs in North Africa (Magnusson 1929). Two species occur in Europe: *A. aeginaica* in the Mediterranean area, lichenicolous on *Aspicilia*, and *A. cinerascens* from the central Alps on sunny lower slopes (Magnusson 1954, 1956; Knudsen et al. 2015). Five species from North America were described: *A. coloradiana*, *A. nevadensis*, *A. peltastica*, *A. peltata*, and *A. utahensis* (Zahlbruckner 1902; Hasse 1911; Magnusson 1929, 1952).

This study of the *Acarospora strigata* group continues our study of the *Acarosporaceae* of southwestern North America (Knudsen 2007; Knudsen et al. 2021a, 2023a, 2023b, 2025a, 2025b).

## Material and methods

### Herbarium study

Specimens were examined from ASU, BRY-C, KRAM, H, hb. H&N (Jason Hollinger and Nastassja Noell), hb. K&K (Jana Kocourková and Kerry Knudsen) and hb. Wheeler (Tim Wheeler). Morphology was studied with dissecting microscopes. The anatomy of hand sections was examined and measured in water at 1000× with compound microscopes. We use a strict protocol for reproducible results for IKI reactions of hymenial gel (Knudsen and Kocourková 2018a; Knudsen et al. 2024). The ascus stain was studied in IKI (Hafellner 1993). The hypothallus was studied by scratching the substrate surface of specimens and examining with dissecting microscopes. Thin layer chromatography (TLC) in solvent C and spot tests according to Orange et al. (2010) were performed on 32 specimens of the species of the *strigata* group species in North America.

### Imaging

Macrophotographs were taken with the digital camera Olympus DP74 mounted on the Olympus SZX 16 stereomicroscope using PROMICRA QuickPHOTO INDUSTRIAL 4 software and stacked using the Olympus DeepFocus 3.5 module for increasing the depth of field. Microphotographs were taken with a digital camera Olympus DP74 mounted on an Olympus BX51 light microscope fitted with Nomarski interference contrast and using PROMICRA QuickPHOTO INDUSTRIAL 4 software. The figure plates were processed with the module figure Maker F7 fitted to the same software.

### DNA extraction, PCR amplification and sequencing

DNA was extracted from 18 dried herbarium specimens (Suppl. material 1 in bold). Genomic DNA was extracted from lichenized thalli via the Invisorb Spin Plant Mini Kit, according to the manufacturer’s protocol with slight modifications (i.e., DNA was diluted to 50 μl instead of the 100 μl recommended by the kit) and incubated in buffer for 15 minutes before final centrifugation. Total extracted DNA was stored at −20 °C. The quality and yield of isolated DNA were checked on a 1% agarose gel and DNA concentration and purity were measured precisely using a UVS‐99 spectrophotometer (ACT Gene). A table of primers are listed in Knudsen et al. (2021b). The selected genes for this study were the nuclear ribosomal internal transcribed spacer ITS: ITS1F: 5'CTTGGTCATTTAGAGGAAGTAA3', ITS4: 5'TCCTCCGCTTATTGATATGC3' (White et al. 1990), the large subunit of the nuclear ribosomal DNA: nLSU; LROR: 5'ACCCGCTGAACTTAAGC3', LR5: 5'TCCTGAGGGAAACTTCG3' (Vilgalys and Hester 1990), and the small subunit of the mitochondrial ribosomal DNA: mtSSU, SSU1: 5'AGCAGTGAGGAATATTGGTC, SSU3R: 5'ATGTGGCACGTCTATAGCCC3' (Zoller et al.1999). The ITS, nLSU, and mtSSU regions were amplified via polymerase chain reaction (PCR). Each reaction contained 1 μl (20–25 ng) of extracted genomic DNA, 10 μl of 2× MyTaq Red DNA Polymerase (Bioline), 8.2 μl of water and 0.4 μM of forward/reverse primer (10 μM) for a total reaction volume of 20 μl. Conditions for ITS and mtSSU rDNA: initial denaturation at 95 °C for 5 min, followed by five cycles (95 °C for 33 s, 56 °C for 30 s and 72 °C for 30 s), then ten cycles (95 °C for 30 s, 54 °C for 30 s and 72 °C for 30 s), and twenty cycles (95 °C for 30 s, 50 °C for 30 s and 72 °C for 30 s), with a final extension at 72 °C for 15 min. For the nLSU: initial denaturation at 95 °C for 1 min, followed by five cycles (95 °C for 30 s, 55 °C for 30 s and 72 °C for 60 s) and finally 30 cycles (95 °C for 30 s, 52 °C for 30 s and 72 °C for 60 s), with a final extension at 72 °C for 10 min. Before sequencing, the PCR products were purified using the enzymatic method ExoSap-IT Express Reagent (Thermo Fisher Scientific, Inc.), according to the manufacturer’s protocol. PCR products were run on a 1.0% agarose gel via electrophoresis and stained with ethidium bromide for 20 min. Purified PCR products, water and forward primer (8 μl total volume) were sent to BIOCEV, Vestec, Czech Republic. Sequences were checked against the UNITE and NCBI databases for contamination. All newly generated sequences were deposited in GenBank (Suppl. material 1).

### Sequence alignment and phylogenetic analysis

We produced new sequences from 18 samples and additional sequences were obtained from GenBank (Suppl. material 1). The sequences were proofread and concatenated manually into a single dataset using SEQUENCHER version 5.4.6. Sequences were aligned using the multiple sequence alignment online service MAFFT version 7.110 with the “G-INS-I” strategy (Katoh and Toh 2008; http://mafft.cbrc.jp/alignment/server/). Indels longer than 1 bp were coded using the simple gap coding method (Simmons and Ochoterena 2000) as implemented in SEQSTATE 1.4.1 (Müller 2005). A partition homogeneity test (incongruence length difference) was performed in PAUP* version 4.0a169 (Swofford 2002) under 1,000 replicates, using a heuristic search to assess congruence among the ITS, nLSU, and mtSSU. The PHT result indicated that incongruence was not significant (P value < 0.1). The combined matrix included a total of 1,596 characters of aligned DNA sequences from mitochondrial and nuclear genes: ITS rDNA (501), nLSU rDNA (619 bp) and mtSSU rDNA (619 bp). We analyzed a combined matrix of the three genes, which included a total of 60 specimens. Of these characters, 1,037 were constant, 225 were variable and parsimony-uninformative and 334 were parsimony-informative. For phylogenetic analyses, the GTR+I model was selected as the best-fitting model of nucleotide substitution for each gene, based on the Akaike Information Criterion using jModelTest 2.1.10 (Darriba et al. 2012). A phylogenetic tree was constructed using MrBayes 3.2.2 (Ronquist and Huelsenbeck 2003). Input data were formatted for MrBayes via the FABOX online converter tool (http://birc.au.dk/~palle/php/fabox/fasta2mrbayes.php; Villesen (2007)). Sequences of *Pycnora sorophora* were included as an outgroup. Three replicate analyses with four chains each were run for 30,000,000 generations, sampling every 1,000^th^ generation. After this number of generations, the average standard deviation of split frequencies reached a value lower than 0.01, indicating convergence. The MCMC analysis of the four concatenated genes was run for 30,000,000 generations. The data was also analyzed using Maximum Likelihood (ML) methods (Suppl. material 2). Three searches for ML analyses were executed under the GTR+GAMMA nucleotide substitution model in RAxML v.8.2.10 (Stamatakis 2014). The Bayesian phylogenetic tree with posterior probability and bootstrap approximation with 1,000 replicates for ML method was visualized using FigTree v.1.4.0 and rooted with *Pycnora sorophora* (Rambaut 2012). The final alignment and list of primers is accessible in the ZENODO database (https://zenodo.org/) under submission https://doi.org/10.5281/zenodo.20303008.

## Results and discussion

**Figure 1.**
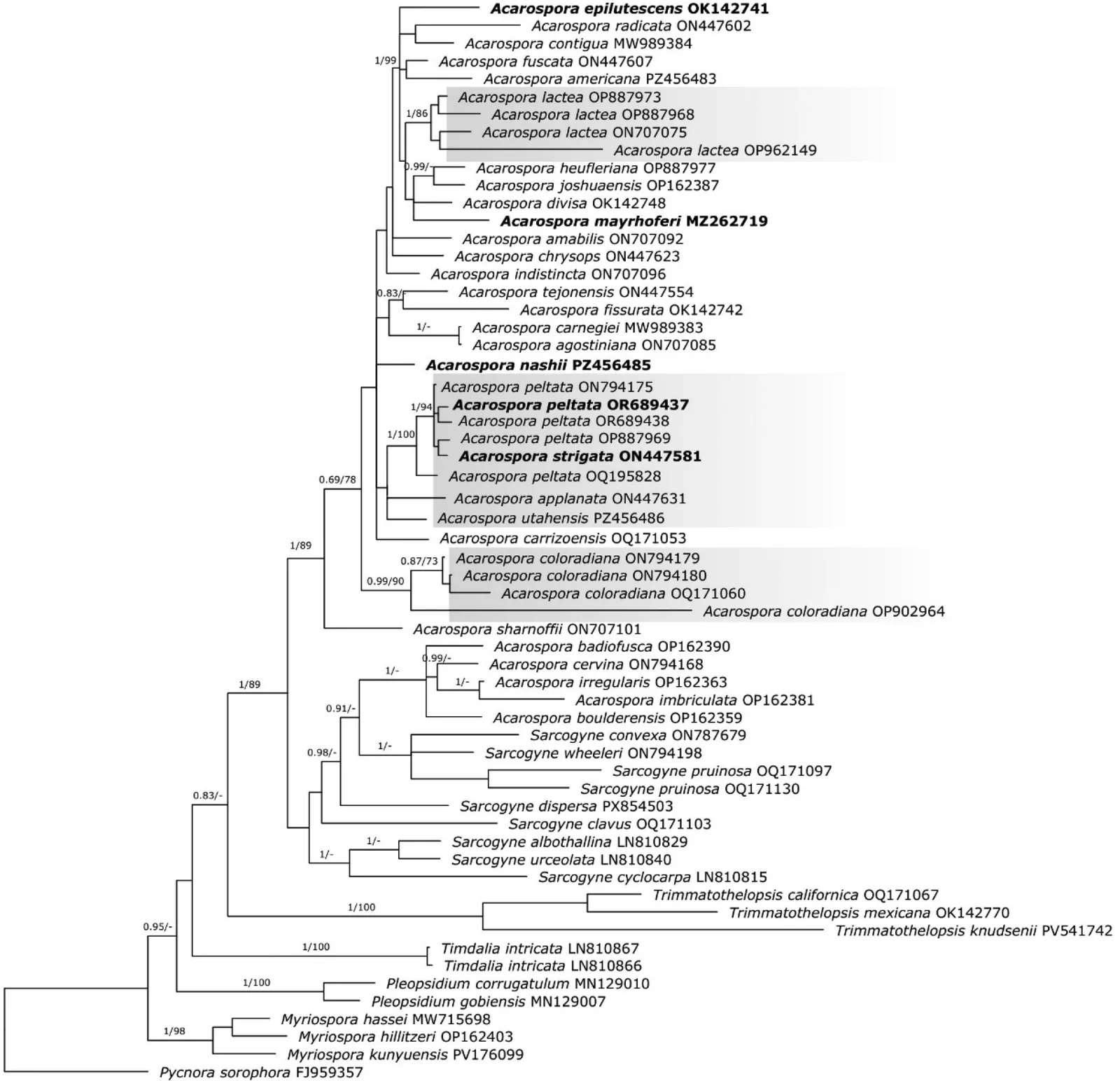
Bayesian inference tree obtained by phylogenetic analysis using a combined data set of ITS, mtSSU, and nLSU sequences of 60 members of *Acarosporaceae*. Bayesian posterior probability (BPP ≥ 95%) and maximum likelihood bootstrap values (ML ≥ 0.70) are indicated above branches (BPP/ML). *Pycnora sorophora* was used as an outgroup.

Our current family phylogeny is congruent with previous phylogenies (Westberg et al. 2015; Knudsen et al. 2021a, 2023a, 2025a, 2025b, 2025c). Current genus names of species have not been changed anticipating further phylogenetic and genomic analysis and wider taxon sampling. In this phylogeny (Fig. 1), all specimens sequenced are from North America and Europe, except for four species from China and one from South America. This is hardly representative of a family which occurs on all seven continents.

In this phylogeny (Fig. 1) we recovered in the upper *Acarospora* clade five members of the morphologically defined *Acarospora strigata* group, *A. strigata* from South America and four species from North America, *A. coloradiana*, *A. lactea*, *A. peltata*, and *A. utahensis*.

*Acarospora peltata* and *A. utahensis* from North America and *A. strigata* from South America are recovered in a *peltata* clade (shaded) which also includes *A. applanata*. *Acarospora utahensis* differs from all other species in having the largest ascospores. It shifts to another clade in the ML tree (Suppl. material 2)*. Acarospora applanata* is not a member of the *A. strigata* group, has a brown epruinose thallus and is a lichenicolous lichen often parasitic on *A. peltata* (Knudsen et al. 2021a). It is also recovered in another clade in the ML tree (Suppl. material 2). *Acarospora strigata* from South America is nested among five specimens of *A. peltata*. We sequenced *A. strigata* (in bold) collected by Valerie Reeb on limestone in Boliva and it has ITS and LSU with a 98% similarity with the epitype of *A. peltata* on caliche, marked in bold (Kocourková 11112a, SBBG)*. Acarospora peltata* and *A. strigata* have a100% similarity with mtSSU. Anatomically, *A. strigata* primarily differs from other *A. peltata* specimens in this clade in having euamyloid hymenial gel and a wider parathecium, both usually species level characters in our taxonomy. We have not discovered if *A. peltata* is a species or species complex. We do not treat *A. strigata* and *A. peltata* as synonyms. For the integrative phylogenetic and taxonomic analysis of the species concepts of *A. strigata* and *A. peltata*, see notes in taxonomic section on *A. strigata*.

In a new lineage, *Acarospora lactea* (shaded) is most closely related to four species sequenced from North America (*A. heufleriana*, *A. divisa* and *A. joshuaensis*) and from Europe (*A. mayrhoferi*).

*Acarospora coloradiana* forms a new lineage (shaded).

Based on this phylogeny (Fig. 1), the *strigata* phenotype, like the phenotype of *A. privigna* (*Polysporina simplex*, nom. illegit.), with carbonized epihymenial accretions, has evolved numerous times and is not recovered in a single clade together (Westberg et al. 2015).

New sequences are published in our phylogeny of *A. epilutescens* (Knudsen 2007), *A. mayrhoferi* (Knudsen and Kocourková 2018b) and *A. nashii* (Knudsen 2011) (in bold).

### Taxonomy

#### Acarospora strigata

Taxon classificationFungiAcarosporalesAcarosporaceae

(Nyl.) Jatta, Malpighia 20: 10 (1906)

A62C2A3B-C750-552A-B368-2AA8CE2FBAE4

375730

Figs 2, 3

≡ Lecanora strigata Nyl., Ann. Sci. Nat., sér. 4, 3: 155 (1855) (P, holotype, n.v.).

##### Type.

Chile, Cordilleris d’Elqui, on porphyry, C. Gay, s.n.

##### Etymology.

*Strigata* describes the fissures which are common in the *strigata* group.

##### Description.

Hypothallus endosubstratal, no algae observed. Thallus composed of squamules or areoles, 0.3–1.5 mm wide, 0.4–0.6 mm thick, scattered or forming patches to 1 cm wide. Upper surface white or bluish gray with pruina, brown when wet, fissures common, but sometimes absent, shallow, sometimes deepening to form abscission fissures for replicating by division. Lower surface white, ecorticate. Epicortex 30–50 µm thick. Eucortex 20–60 µm tall, cells paraplectenchymatous, 2–4(–6) µm wide, upper layer reddish brown, lower layer hyaline. Algal layer even, 80–100 µm thick, continuous and thinner under apothecia, algal cells 8–12 µm wide. Medulla 200–400 µm thick, white, obscure in water, thin-walled hyphae 3–5 µm wide, branching, coated often with crystals. Apothecia one to four per areole or squamule, immersed to rarely pseudo-lecanorine, disc usually round, reddish brown to black, epruinose, punctiform to 1 mm in width. Parathecium of thin hyphae, IKI-, expanding to 40–80 µm wide around disc, sometimes visible as parathecial ring. Epihymenium reddish-brown, 15–20 µm tall. Hymenium 100–120(–140) µm tall, IKI+ dark blue, euamyloid (using our protocol). Paraphyses 1.5–2.0 µm wide at mid-level, apices 3–4 µm in a gel cap. Asci 70–110 × 10–20 µm, cylindrical to clavate, ascospores 100–200 per ascus. Ascospores 3–5(–6) × 2.0(–2.5) µm (n = 50+). Subhymenium 40–50 µm tall, IKI+ blue. Hypothecium 10–15 µm tall, IKI-. Pycnidia rare, conidia 1–2.5 × 0.5–1 µm. Not producing secondary metabolites.

**Figure 2.**
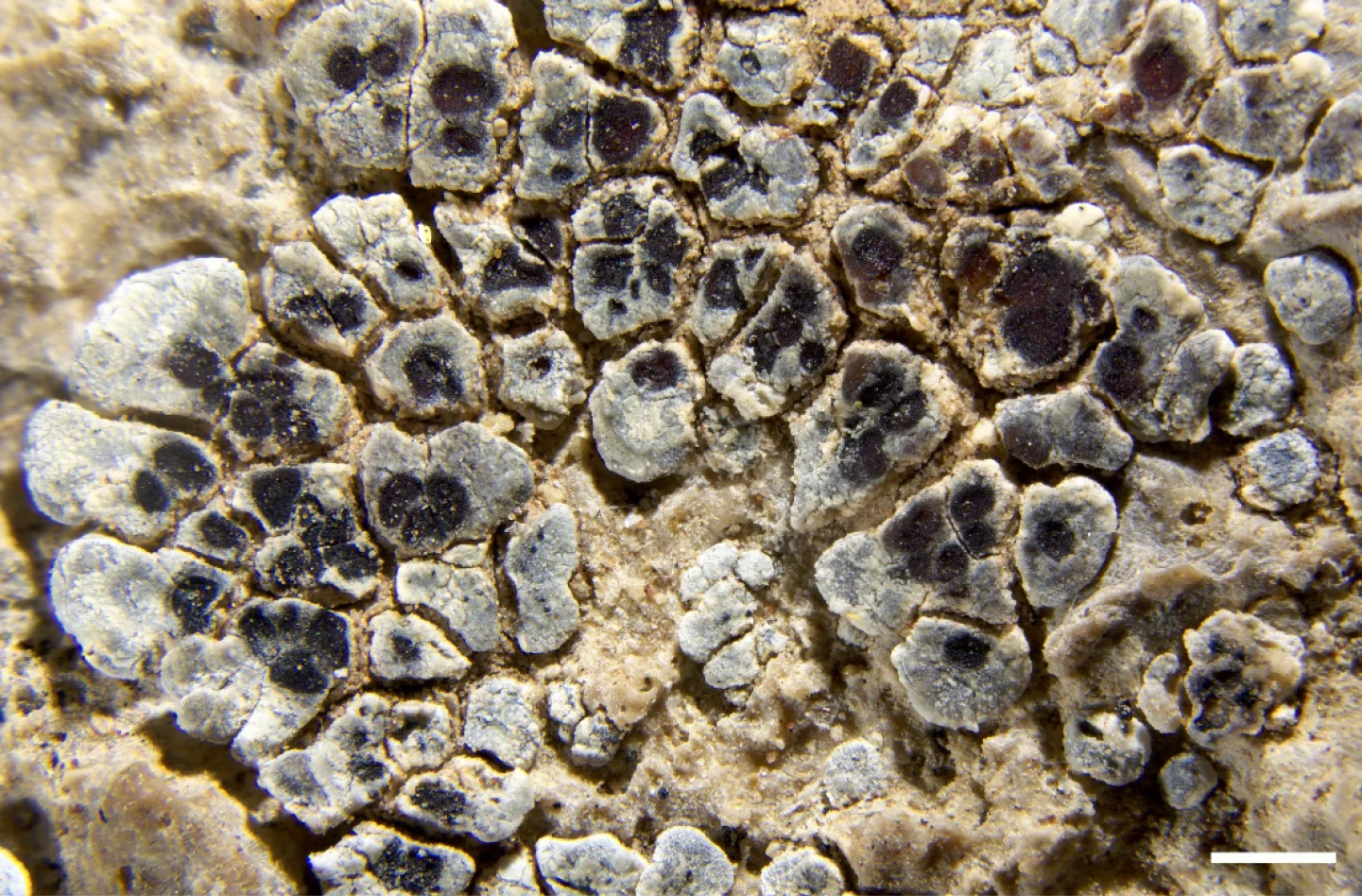
*Acarospora strigata*, Boliva, V. Reeb 15-V 2 /2 (hb. K&K). Habit of the thallus with apothecia. Scale bar: 1 mm.

##### Habitat and distribution.

In South America, in the Andes (Argentina, Bolivia, Chile) on calcareous and non-calcareous rock (Magnusson 1929). Reports of the species are rare and in need further study.

##### Specimens of *Acarospora strigata* from South America examined.

• Argentina, Dept. Mendoza, Lugan, Potrerillos and Cacheuta, alt. 1110 m, on HCl- rock, 18 Jun. 1939, A. Ruiz Leal 6479 (H); • Las Heras, Cerro de la Gloria, no elevation, on HCl- rock, 18 Jun. 1939. A. Ruiz Leal (H); • Cerro de la gloria, alt. 1100 m, on HCl+ rock, 8 Jun. 1939, A. Ruiz Leal (H); • Bolivia, Dept. Potosi, Prov. Daniel Campos, Salar Uyumi, Isla del Incahuasi, on limestone, alt. 3667 m, -20.2408, -67.6275, 3 Dec. 2009, A. Flakus 14665, 14677, 14668 & P. Rodriguez (KRAM); • At the foot of Tunupa volcano, W of the Village of Coquesa, along the Salar of Uyuni, alt. 3702 m, on calcareous rock, 15 May 2002, V. Reeb 15-V 2 /2 (NY, SBBG, hb. K&K); • Chile, Coquimbo, on calcareous rock, Claude Gay s.n. (H-NYL 24877, H-NYL 24898, H-NYL 3317).

##### Notes.

It should be noted that the description of *Acarospora strigata* in Magnusson (1929) is not a description of *A. strigata* from South America. Magnusson wrote: “*A. strigata* is a very variable species both in structure and in habitat, but I have examined more than a dozen squamules from different specimens without finding a definite correlation between substratum and characters or between different groups of characters. It is impossible therefore to make a further division”. His description is a composite, based on four specimens from South America, at least seven specimens from North America (California, Colorado, Wyoming), two from Egypt, one from Iran and probably six from Siberia. We counted at least 20 specimens he studied (Magnusson 1929).

Our taxonomic analysis and description of *A. strigata* was based on 10 specimens from South America (Argentina, Bolivia, Chile). While we could not see the type in Paris collected by Claude Gay and determined by Nylander, we were able to study three collections by Gay and determined by Nylander from Chile. *Acarospora strigata* differed from *A. peltata* in size of areoles or squamules 0.3–1.5 mm vs. (0.3–)1.0–2.0(–3.0) mm wide. Ascospores of *A. strigata* differed from *A. peltata*, 3–5(–6) × 2(–3) vs. (2.0–)4.0–5.0(–8.5) × 1–3 μm (n = 50+) and lacked the variability of ascospore sizes common in *A. peltata*. *Acarospora strigata* had a wider parathecium than *A. peltata*, 40–80 vs. 10–40 μm wide and sometimes indistinct. We consider the width of parathecium to be an informative character. The epicortex of *A. strigata* had a thickness of 30–50 µm vs. up to 110 µm in *A. peltata*.

*Acarospora strigata* also differed from *A. peltata* in having euamyloid hymenial gel vs. hemiamyloid hymenial gel, a species level character in *Acarosporaceae* in our taxonomy. Nylander describes the holotype as having hymenial gel IKI+ red (Nylander 1855) There was no protocol for repeatable results for the I used in 19^th^ century or by Magnusson. For instance, some formulas with larger amounts of I can produce hemiamyloid results (various shades of red). Though we could not test or view the holotype, all the tests we did on *A. strigata* were euamyloid using our protocol for repeatable results [see protocol for reproducible results in the introduction of key in this paper or Knudsen and Kocourková (2018a) and Knudsen et al. (2024)]. It is possible that there is a second species in South America with IKI+ hemiamyloid hymenial gel.

In our phylogeny, *Acarospora strigata* was recovered in a clade with five specimens determined as *A. peltata*. For the reasons given above, we do not treat them as synonyms. We have not discovered if *A. peltata* is a species or species complex. In this paper, *A. strigata* is treated as a separate species from *A. peltata* and endemic to South America.

**Figure 3.**
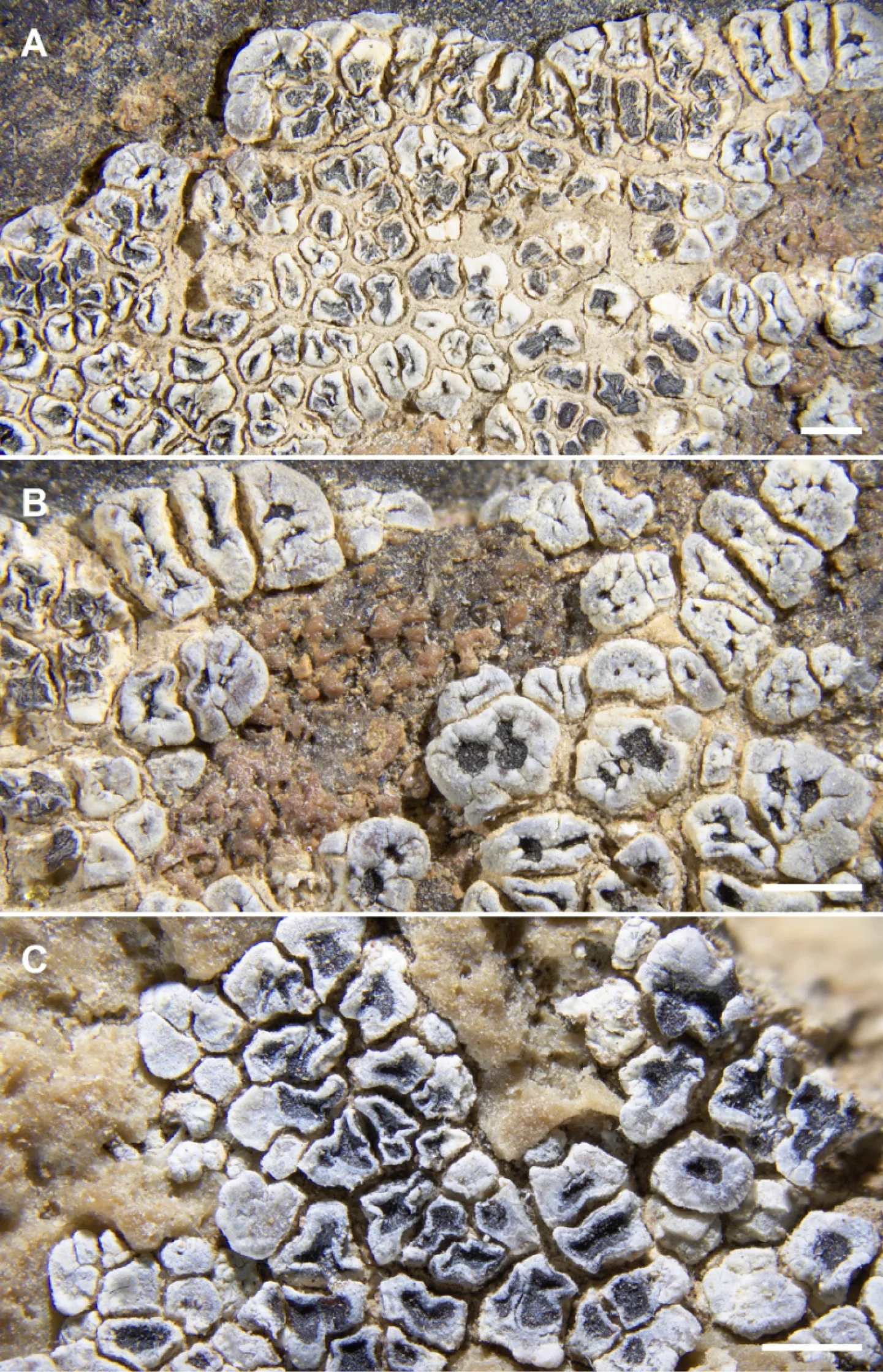
*Acarospora strigata*, s. lato. Asia. Golubkova 287, Mongolia (**A, B**), Golubkova 1063, Tajikistan (**C**). **A**. Habit of the thallus with apothecia, thallus developing on mycelial base; **B**. Areoles with 1–3 apothecia replicating by division; **C**. Habit of the thallus with apothecia. Scale bars: 1 mm (**A–C**).

Specimens Magnusson identified as *Acarospora strigata* in China also need further study (Magnusson 1940, 1944). Specimens determined as *A. strigata* with hemiamyloid hymenial gel reactions are common in Xinjiang (Nurtai et al. 2017). In a taxonomic study of 38 specimens of Chinese *A. strigata*, its appearance looked like a typical *A. peltata* morph with similar ascospores 3.0–7.5 × 2.0–3.5 μm, but it had a hymenium 80–120 μm high, a lower range than *A. peltata* (Nurtai et al. 2017). It has a parathecium 22–40 μm wide corresponding to the width of parathecium in South American *A. strigata*. Golubkova collected similar specimens in Mongolia and Tajikistan (Fig. 3).

Recently, a new species of the *Acarospora strigata* group, *A. iqbalii*, was published from Pakistan (Iqbal et al. 2025). It has a parathecium 10–25 μm thick, a hymenium 50–100 μm tall, hymenial gel IKI+ blue turning red, hemiamyloid and ascospores 3–4 × 2–3 μm. *Acarospora iqbalii* was recently discovered in China (Liang et al. 2026).

Magnusson (1940) reported seven collections by Bohlin of *A. strigata* on calcareous rock in Kansu, China, four with small globose ascospores, one with ascospores 5–8 × 2.0–2.5 μm and two with ascospores 4–5 × 1.7 μm, none with IKI reactions of the hymenial gel or other descriptions. He compared them to *A. peltata* which may just mean the areoles were large. We have not studied these specimens, but, based on ascospore size, they represent more than one species.

##### Specimens of *Acarospora strigata* s. lato from Asia examined.

Mongolia, northern edge of the Galbyn-Gobi Desert, north-east of Temen-Chuluu, on destroyed granite, 2 Aug. 1970, N. S. Golubkova, Tsogt, 1063 (H); • Tajikistan, Gorno-Badakhshan Mountainous Autonomous Region, Murghob Distr., Eastern Pamir, Murghab Dist. Near the Chechekty River, high mountain cold desert, alt. 3860 m, rocky slope on small silicate stones at ground level, 15 Jul. 1965, N. S. Golubkova 287 (H).

#### Acarospora peltata

Taxon classificationFungiAcarosporalesAcarosporaceae

Hasse, The Bryologist 14 (1): 3 (1911)

EEEADE10-92F6-5FDD-A1D7-AC65C67968DB

375666

Figs 4, 5

= Acarospora strigata f. longispora H. Magn, syn. nov. K. svenska Vetensk-Akad. Handl., Ser. III 7(no. 4): 208 (1929). Type: U.S.A. • Colorado, bei der station Kingmann, 1905, Rechinger (W, n.v.).

##### Type.

U. S. A., Arizona, near Ademana, Jun. 1904, Hasse (FH! holotype).

**Figure 4.**
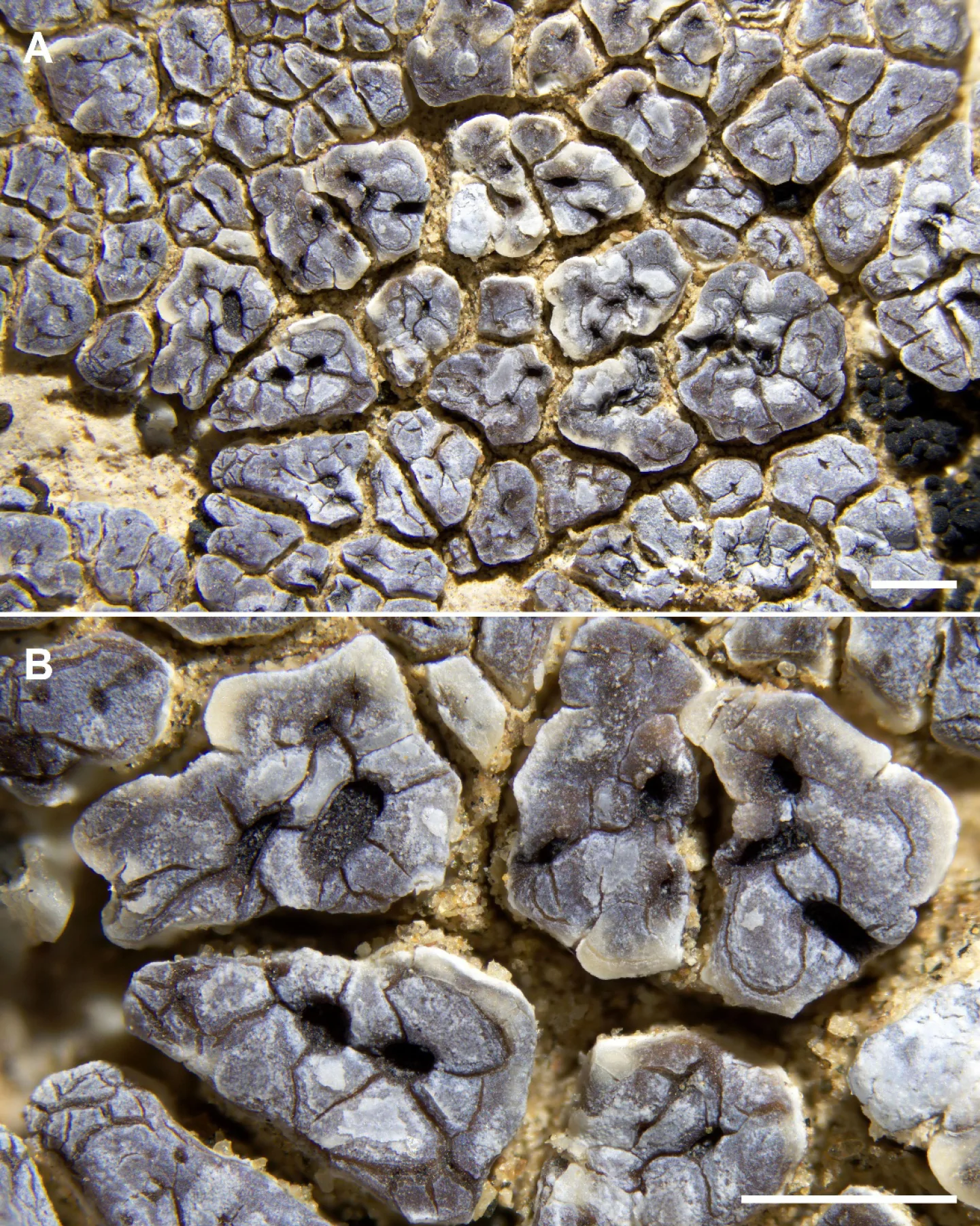
*Acarospora peltata* Kocourková 11112a, epitype (**A, B**). **A**. Habit of the areolate thallus with apothecia; **B**. Detail of areoles with immersed apothecia. Scale bars: 1 mm (**A, B**).

**Figure 5.**
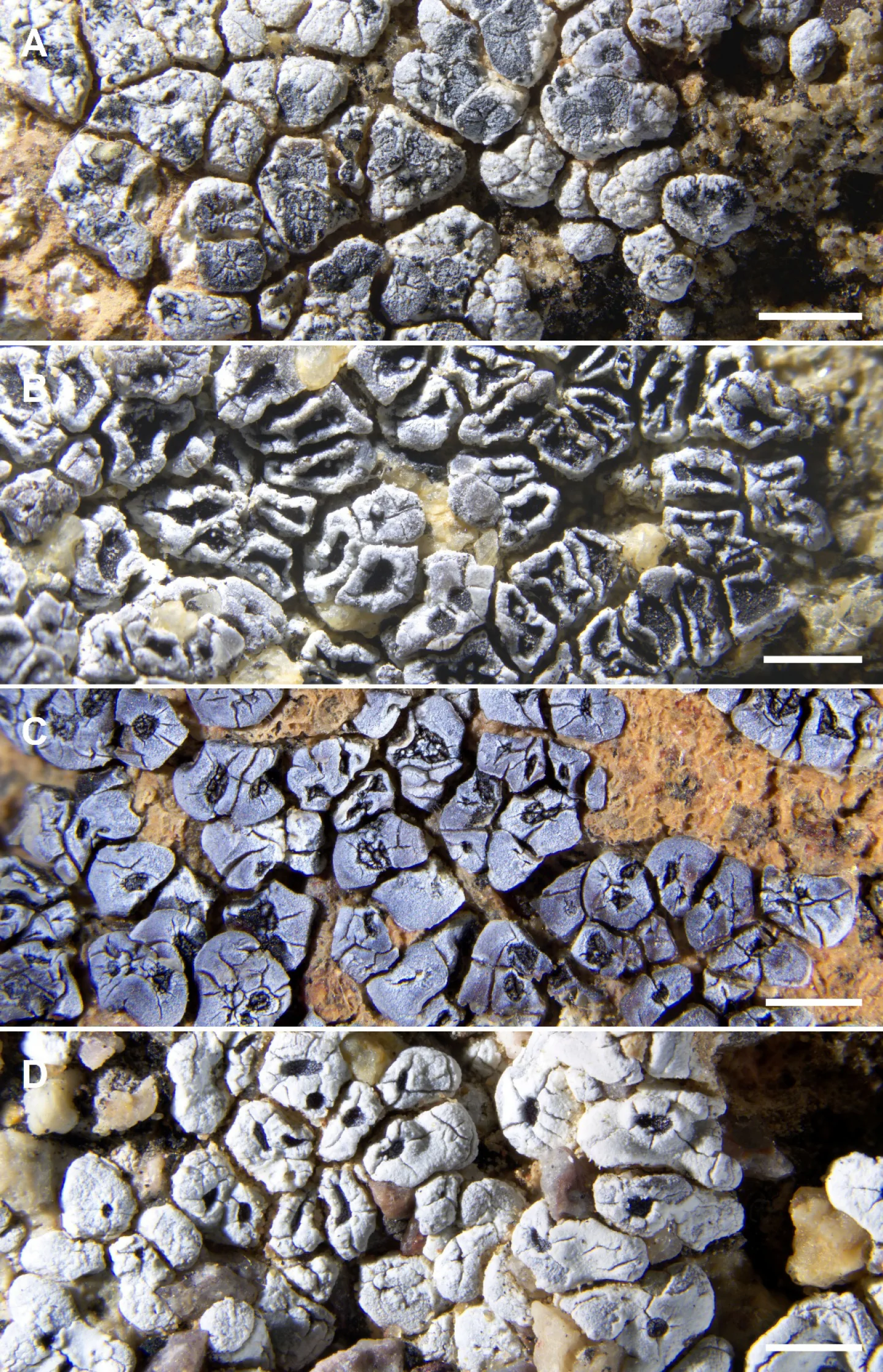
*Acarospora peltata*, Wheeler, 1936 (**A**); Kocourková 11114c (**B**); Hollinger 16148 (**C**); Holliger 16602 (**D**). **A–D**. Variability of the species. Habit of the areolate thallus with apothecia. Scale bars: 1 mm (**A–D**).

##### Epitype.

U.S.A., California, Riverside Co., Joshua Tree National Park, Sonoran Desert, low unnamed crest between Smoke Tree Wash and Pinto Basin Road, 33.9463, -115.968, alt. 950 m, at base of west facing slope, on caliche, annual flooding, 28 Mar. 2023, J. Kocourková 11112a & K. Knudsen, epitype (and reference genome) and isoepitype, MBT 10033394 (SBBG).

##### Etymology.

Named for the distinct mycelial base or stipe uplifting well-developed squamules.

##### Description.

Hypothallus endosubstratal, no algae observed. Thallus dispersed or contiguous, rarely producing lobes around the margins (which eventually split into small areoles by replication by division), up to 10 cm wide. Areoles or squamules (0.3–)1.0–2.0(–3.0) mm wide, (0.2–)0.35–0.5(–1.0) mm thick, flat to convex, broadly attached to substrate or with a mycelial base, sometimes narrowing to a stout stipe or sometimes the mycelial base forming an ecorticate epilithic thallus spreading between and under numerous apothecia. Upper surface white pruinose, sometimes with bluish or gray tint, usually with fissures, radial around apothecia or crosshatched, dense or not, shallow or cleaving through the epicortex down to the eucortex, translucent when wet, turning brown (from eucortex below) or, if epicortex or pruina, is very thick, turning a subtle grayish white or remaining white. Lower surface white or darkened by substrate interactions, ecorticate. Epicortex a polysaccharide matrix, sometimes with hyphae 0.5 µm thick visible running horizontally to the cortex, the upper layer mixed with pruina, varying from 10–110 μm thick. Eucortex 25–65 µm thick, upper layer reddish-brown, 10–20 µm thick (rarely absent), lower layer hyaline, cells round to irregular 2–3(–5) µm wide. Algal layer 50–250 μm thick, continuous or not under apothecia, sometimes interrupted by occasional bands of a hyphae 10 μm wide, algal cells usually 10–12 μm wide. Medulla 100–500 μm thick, obscure, hyphae encrusted with substrate crystals, 2–5 μm wide, thin-walled. Apothecia often 1 per areole, rarely up to 8, sometimes, in poor microhabitats, many areoles lacking apothecia, punctiform to 2 mm wide, disc immersed, usually black, reddish-brown or black when wet, lightly pruinose or not, sometimes reducing the areole or squamule to a thalline margin. Parathecium 10–40 μm wide, hyphae 1–2 μm wide, but sometimes hard to distinguish from hymenium, IKI-, sometimes forming a thin ring around the apothecia, sometimes slightly higher than disc. Hymenium (100–)110–150(–180) μm tall, reddish-brown, epihymenium ca. 10 μm tall, paraphyses 1.0–2.0(–2.5) μm, sometimes branching, apices not expanded in a thin gel cap 2–3 μm wide, often with darker reddish outline, hymenial gel IKI+ red or blue turning red in squash preparations, hemiamyloid. Asci 90–140 × 10–50 μm, ascospores variable, sometimes in same hymenium, ellipsoid to broadly ellipsoid, (2.0–)4–6(–8.5) × 1–3 μm (n = 50+). Subhymenium 20–30 μm tall, sometimes V- or U-shaped, 50–80 μm tall, IKI+ dark blue. Hypothecium 10–20 μm tall, sometimes V- or U-shaped surrounding subhymenium, hyphae 1–2 μm, IKI-. Pycnidia not observed. Not producing secondary metabolites; UV-, but sometimes UV+ yellow or orange which is caused by crystals of calcium oxalate monohydrate (the mineral whewellite), which is one of the most common oxalates found in lichens (Knudsen 2007; Krajanová 2023; Knudsen et al. 2026).

##### Habitat and distribution.

U.S.A. (Arizona, California, Colorado, Kansas, Montana, Nevada, New Mexico, South Dakota, Texas, Utah, Wyoming) on limestone, caliche, granite, gneiss, HCl-/+ sandstone, rhyolite, lava, usually in full sun, in desert and arid habitats at a variety of elevations, but largest specimens occur in canyons with intermittent humidity or regular water and shade part of the day. Specimens from Mexico were examined in a previous study (Chihuahuan and Sonoran Regions, Baja California) (Knudsen 2007). Its southern limit in Mexico is unknown. The abundance of the white *strigata* group and yellow species of *Acarosporaceae* are a distinctive character of the lichen flora of North American deserts.

##### Additional specimens examined.

U.S.A. • California, Riverside Co., Joshua Tree National Park, Little San Bernardino Mountains, Mojave Desert, Eureka Peak, below summit, -20.5847, 15.233, alt. 1670 m, 28 Mar. 2023, J. Kocourková 11114c & K. Knudsen (SBBG); • San Jacinto Mountains, Sonoran Desert, Pines to Palms Highway, 33.5817, -116.6347, alt. 1223 m, low crest on caliche, 24 Mar. 2023, J. Kocourková 11115a, 11115b, 11115c & K. Knudsen (hb K&K); • San Bernardino Co., San Bernardino Mountains, Rattlesnake Canyon, 34.2310, -116.6601, alt. 1803 m, on granite, 10 Oct. 2014, K. Knudsen 17234; • 34.2458, -116.6491, alt. 1769 m, 12 Oct. 2014, K. Knudsen 17221 (hb. K&K); • Montana, Besferhead Co., Highway 342, 44.9917, -112.8761, alt. 1768 m, on Madson limestone, 17 May 2011, T. Wheeler 3412 (hb. Wheeler); • Choteau Co., Ceceiele Park, near Iuad, 47.7842, -109.7816, alt. 992 m, on Eagle Sandstone, 15 Jul 2011, T. Wheeler 3453 (hb. Wheeler); • Carter Ferry, Missouri River Breaks, 47.7523, -110.9065, alt. 853 m, on sandstone cobbles, 2 May 2011, T. Wheeler 3390, 3450 (hb. Wheeler); • Meagher Co., Highway 294 near Lennep, 46.4119, -110.5515, 5290 m, 5 Jan. 2015, T. Wheeler 7349 (hb. Wheeler); • Nevada, Clark Co., Echo Wash, American Borax Road, 36.2918, -114.5294, alt. 548 m, on red HCl- sandstone, 30 Mar. 2009, T. Wheeler 2658 (hb. Wheeler); • Lincoln Co., Antelope Canyon, top of limestone cliffs on N side of canyon, ca. 0.75 mi. W of Hwy. 93, 37.638, -114.52, alt. 1550 m, 30 Mar. 2016, N. Noell 1041, 1550 & J. Hollinger (BRY-C); • Condor Canyon, on shaded rhyolite, 37.832, -114.359, alt. 1510 m, on rhyolite, 6 Jun. 2016, J. Hollinger 15980 & N. Noell (BRY-C); • Golden Gate Range, Cottentail Pass, open pinyon-juniper woodland, at foot of tall N-facing limestone cliff, 37.951, -115.392, alt. 1700 m, on limestone ledge, 6 Jun. 2016, J. Hollinger 10966 & N. Noel (BRY-C); • Bunker Hills, ca. 7 km W of Meadow Valley Wash in wash ca. 3 km S of Nox, 36.858, -114.747, alt. 900 m, open, blocky S-facing rhyolite outcrop several meters above a calcareous wash, on sunny ash-tuff, 8 Apr. 2017, J. Hollinger 16148 & N. Noell (BRY-C); • Terry Benches, ca. 20 km N of Mesquite, 36.989, -114.086, 800 m a.s.l., N-facing limestone cliff line at top of very loose, open slope, on calcareous sandstone pebble, 18 Apr 2017, J. Hollinger 16602 & N. Noell (BRY-C); • Highland Peak, ridge to west of western peak, 37.897, -114.587, alt. 2835 m, 2 Jun. 2016, J. Hollinger 12648 & N. Noel (BRY-C); • Toquop Wash, 36.88, -114.175, alt. 730 m, 14 Apr. 2017, J. Hollinger 16344a, b & N. Noell (BRY-C); • Worthington Range, hill north end of range, w-side of divide, 37.957, -115.606, alt. 2050 m, 30 May 2016, N. Noel 2370 & J. Hollinger (BRY-C); • Nye Co., Humboldt-Toiyabe National Forest, vic. of Currant Mountain Wilderness Area, at Broom Canyon, 38.8897, -115.4994, alt. 2063 m, 29 Oct. 2016, S. Leavitt 16680, (BRY-C); • White Pine Co., Great Basin National Park, along South Fork of Big Wash, 38.8783, -114.2170, alt. 2377 m, 18 Jul. 2017, S. Leavitt 17559, 17560, (BRY-C); • New Mexico, Eddy Co., Chihuahuan Desert, Brokeoff Mountains Wilderness Study Area, 32.2056, -104.8418, alt. 1852 m, on limestone, 27 Mar. 2022, K. Knudsen 19458 & J. Kocourková (hb. K&K); • Lincoln Co., Chihuahuan Desert, Carrizozo Malpais, Valley of Fire Recreation Area, on HCl+ sandstone slope above lava beds, 33.6839, -105.9393, alt. 1590 m, 19 Mar. 2020, K. Knudsen 19355 & J. Kocourková (hb K&K), • 33.6839, -105 22.04, 1616 m, on sandstone outcrop, 22 Mar. 2020, K. Knudsen 19072 & J. Kocourková (hb. K&K), K. Knudsen 19386 (hb K&K); • Utah, Carbon Co. Helper Water Tower, Gun Club Road, 39.6834, -110.8648, alt. 1981 m, on sandstone, 3 Apr. 2011, T. Wheeler 3220, 3222 (hb. Wheeler); • Grand Co., Moab, Cedar Ridge, 38.6898, -109.6887, alt. 1379 m, 4 Apr. 2016, T. Wheeler 7215 (hb. Wheeler); • Tusher Canyon, Book Cliffs, 34.0992, -110.0319, alt. 1420 m, on HCl- sandstone, 4 Apr. 2011, Tim Wheeler 3232 (hb. Wheeler); • Emery Co., San Rafael Desert, vicinity of Three Canyons overlook, on rim west of the Green River, 38.7087, -110.1245, alt. 1320 m, on sandstone, 15 Apr. 2023, S. Leavitt 23062, 23063, (BRY-C); • San Rafael Swell, Buckhorn Wash, at mouth Calf Canyon, on sandstone escarpments, 39.107, -110.669, alt. 1600 m, 24 May 2024, S. Leavitt 24133, 24138, 24144b (BRY-C); • San Rafael Swell, head of Crack Canyon; 38.633, -110.740, alt. 1720 m, Mar. 2017; S. Leavitt 17001 (BRY-C); • Garfield Co., Dixie National Forest, Upper Sand Creek Research Natural Area, 37.9268, -111.5966, no elevation, 15 Oct. 2021, S. Leavitt 21395 (BRY-C); • Juab Co., along State Route 132, about 14.5 miles west of Nephi, on south side of the road in juniper woodland, 39.649, -112.079, alt. 1650 m, on Fernow Quartz Latite, 21 Oct. 2023, S. Leavitt 23147 (BRY-C); • vicinity of Topaz Mountain amphitheater, on rhyolite rocks in soil, 39.685, -113.075, alt. 1651 m, on rhyolite rocks in soil, 21 Oct. 2023, S. Leavitt 23314 (BRY-C); • Kane Co., south of Kanab, along Landfill Road, in pinyon-juniper woodland, 37.001, -112.517, 1500 m, on Holocene to Upper Pleistocene alluvial gravel, 10 May 2024, S. Leavitt 24063 (BRY-C); • along the Tilted Mesa trail system (Bureau of Land Management), ca. 13 miles east of Kanab. Chinle Formation and Moenkopi Formatio 37.021, -112.351, 1580 m, on sandstone ledges, 10 May 2024, S. Leavitt, 24049 (BRY-C); • Millard Co., Ice Springs cinder cone, 38.9328, -112.5040, 1430 m, on young volcanic rock less than 1000 years old, 27 Dec. 2017, S. Leavitt 17-764 (BRY-C); • Sevier Co., Willow Creek, 38.7242, -111.3078, alt. 808 m, on Ferron sandstone, 5 Apr. 2011, T. Wheeler 3299 (hb. Wheeler); • Washington Co., along Lytle Preserve Ranch Road, 37.1160, -113.9934, no elevation, HCl+ sandstone, 13 Mar. 2009, G. Shrestha GS-017 (BRY-C); • Wasatch County Uinta geocline, Currant Creek Basin, on sandstone, 22 Jun. 1990, Clayton Newberry 1009 (BRY-C, not accessioned); • lower part of Uinta geocline, east of Herber, south-facing gentle slope of Oquirrh sandstone, 3 Jul. 1990, C. Newberry 1012 (BRY-C); • Wyoming, Sweetwater Co., Flaming Gorge National Recreation Area, 41.3516, -109.4194, 1890 m, 5 Sept. 2016, S. Leavitt 16583 (BRY-C); • Firehole Overlook, 41.4041, -109.3068, 2179 m, 17 Mar. 2017, on sandstone T. Wheeler 7382 (hb. Wheeler).

##### Notes.

Similar to *Acarospora peltastica* with hemiamyloid hymenial gel and a thick pruinose epicortex with deep fissures, but differing especially in having ascospores ellipsoid or broadly ellipsoid, (2.0–)4–6(–8.5) × 1–3 μm vs. globose to oval ascospores (3.5–)4–5 μm. They have a sympatric distribution in southern California.

*Acarospora peltata* has phenotypic plasticity and varies in appearance, based on microhabitats, for instance, slope aspect, rock type (often smaller on very hard rock), in full sun all day or partially shaded, in open desert or in canyons or washes, different elevations, amount of annual rain etc. Unfortunately, this information is not usually on labels. The largest areoles were 2.5–3 mm wide, with deep fissures, but not usually becoming subdivided, with several apothecia and with the highest hymenia. They are usually found in washes or canyons with partial shade, flooding during rainstorms or even regular seasonal streams fed by higher elevations. The holotype is a large specimen from near Ademana, in the Sonoran Desert in Arizona (Hasse 1911; Magnusson 1929). The epitype of *A. peltata* is from the Sonoran Desert in Joshua Tree National Park, on seasonally flooded caliche, in Riverside County, southern California.

We think these large specimens are just one extreme of the spectrum of phenotypes. On the other extreme of the spectrum are tiny specimens with no apothecia or punctiform apothecia and short ascospores growing on hard limestone or acidic or basic boulders in the desert in full sun with only water from rare summer or winter rain. Due to this phenotypic variation, Hasse often wrote on his packets of smaller specimens *subpeltata*, an unpublished name (Knudsen 2007).

We have not yet resolved *Acarospora peltata* as a species or species complex. In this paper, we treat *A. peltata* as one species and the common species in western North America.

#### Acarospora coloradiana

Taxon classificationFungiAcarosporalesAcarosporaceae

H. Magn., Kungl.-Akad. Handlingar 7, 4: 273 (1929)

053007C1-1A79-596A-8505-818B3D736549

375522

Figs 6, 7

##### Type.

U.S.A., Colorado. H. Willey s.n., no date (W! holotype).

**Figure 6.**
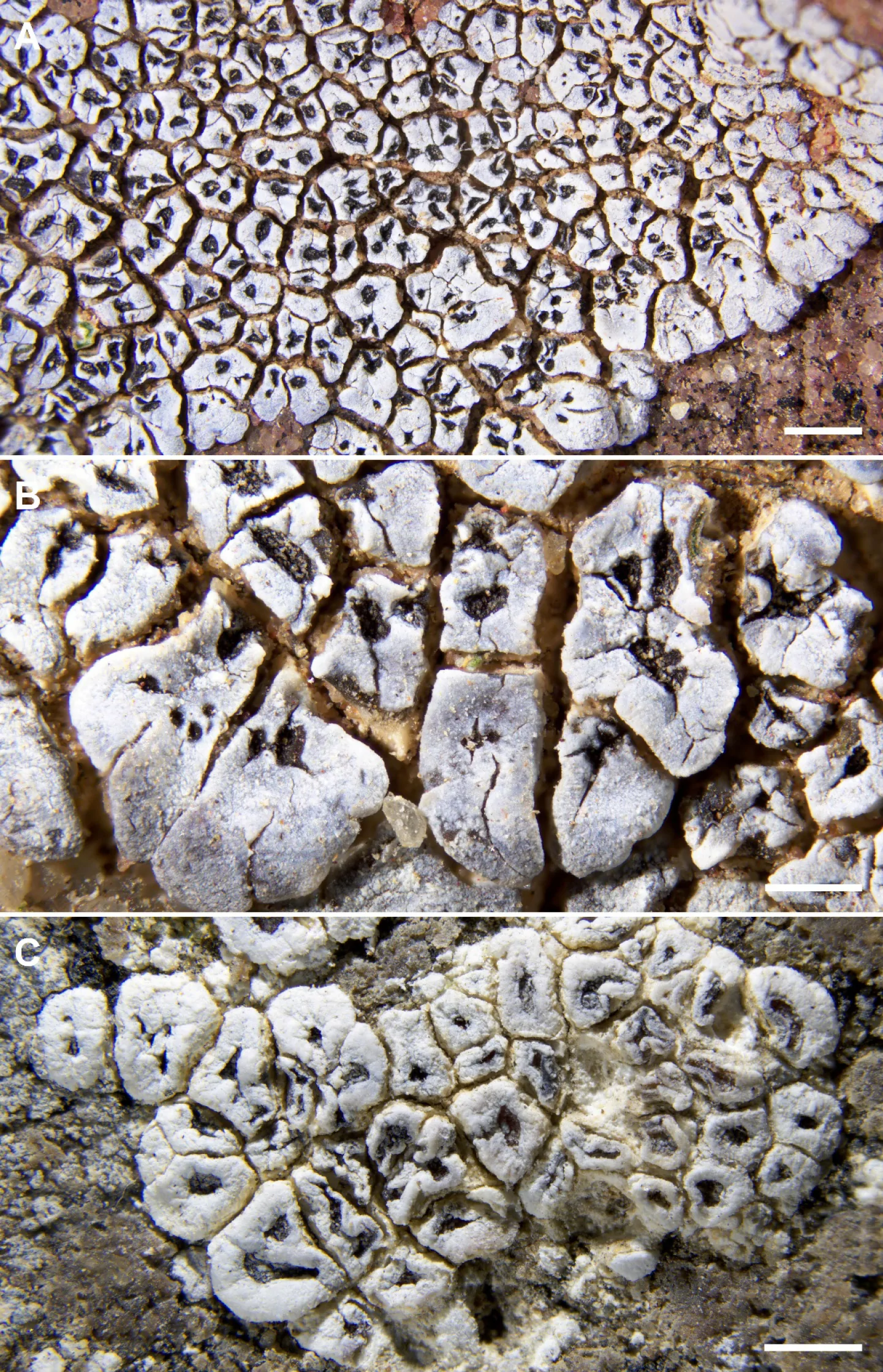
*Acarospora coloradiana*, Kocourková 10687 (**A, B**), Wheeler 3450 (**C**). **A**. Habit of the thallus with apothecia; **B**. Part of the thallus with prolongated marginal areoles; **C**. Habit of the thallus with apothecia. Scale bars: 1 mm (**A, C**); 500 µm (**B**).

**Figure 7.**
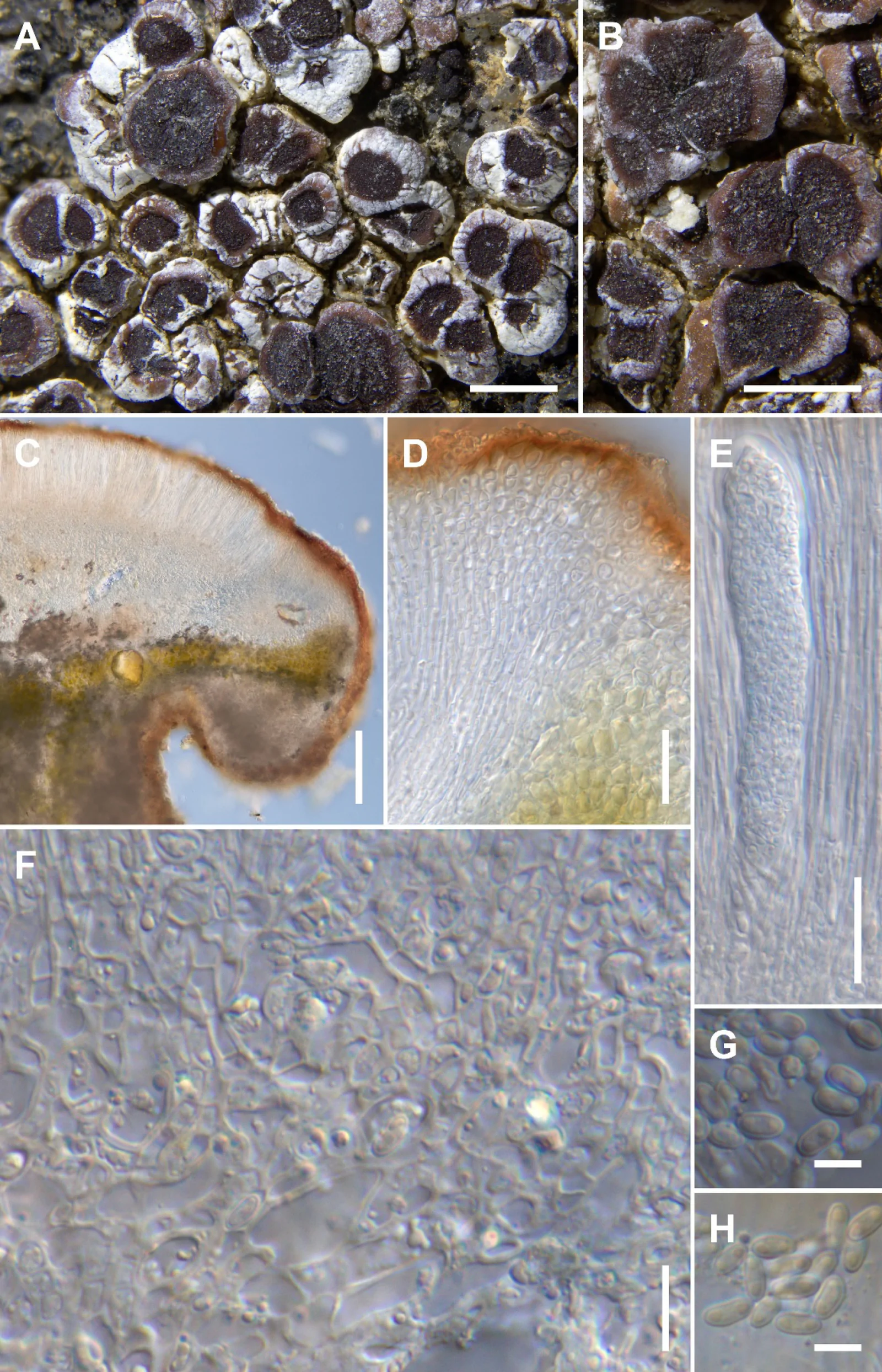
*Acarospora lactea*, Holotype. **A**. Stipitate squamules with apothecia; **B**. Detail of apothecia; **C**. Vertical section of stipitate apothecium showing high hypothecium; **D**. Parathecium; **E**. Young ascus; **F**. Subhymenium of intricate hyphae with some swollen cells; **G, H**. Ascospores. Scale bars: 1 mm (**A, B**); 100 μm (**C**); 20 μm (**D, E**); 10 μm (**F**); 5 μm (**G, H**).

##### Description.

Hypothallus endosubstratal, no algae observed. Areoles or squamules, dispersed or contiguous, (0.2–)1.0(–3) mm wide, (0.1–)0.35–0.5 mm thick, replicating by division, broadly attached or with a mycelial base or narrowed to a stipe, sometimes mycelial base epilithic and ecorticate, continuous between numerous areoles or squamules, flat to convex, sometimes areole or squamule reduced to margin around apothecia. Upper surface white pruinose, usually translucent when wet, brown or gray, rarely white with thick epicortex and eucortex, with or without fissures, replicating by division. Epicortex of polysaccharide matrix and pruina, 10–110 μm thick, often continuous. Eucortex 20–40 μm thick of hyphae 1–2 μm wide running horizontally or more often disarticulated round to irregular cells 2–4 μm wide, upper layer reddish-brown, lower layer hyaline, rarely both hyaline. Algal layer 50–250 μm thick, algal cells 10–12 μm wide, round, oblong to irregular, continuous or not under apothecia, sometimes interrupted by bands of hyphae 10 μm wide. Medulla 100–300 μm thick, obscure with substrate crystals, hyphae 1–2 μm wide, usually encrusted with crystals. Apothecia usually 1–5 per areole or squamule, punctiform to 0.7 mm wide, disc immersed, usually lightly pruinose, reddish-brown when wet, sometimes reducing an areole or squamule to a thalline margin. Parathecium 10–40 μm wide, hyphae 1–2 μm wide. Hymenium 100–140(–150) μm, red-brown epihymenium ca. 10 μm tall, paraphyses 1.0–2.0 μm wide, sometimes branching, apices not expanded in a thin gel cap 2–3 μm wide often with darker reddish pigment mark, hymenial gel IKI+ dark blue, stable or the same hymenium dark blue becoming partially dark and brown, rarely turning partly red in squash preparations particularly from very small apothecia, but never completely red and hemiamyloid. Asci 90–140 × 20–50 μm, ascospores globose to broadly ellipsoid, 2–3 × 2–4 μm, rarely thin ellipsoid, but, in a maturing ascus, always small and globose. Subhymenium 10–20 μm tall, IKI+ blue. Hypothecium indistinct to 20 μm tall, hyphae 1–2 μm wide, IKI-. No pycnidia observed. Not producing secondary metabolites.

##### Habitat and distribution.

In U.S.A., in California, Colorado, Montana, Nevada, New Mexico and Utah on calcareous (limestone, sandstone) and non-calcareous rock. Expected in adjoining States and in northern Mexico. Sometimes growing with *Acarospora peltata*, for instance, in the Tularosa Basin in the Chihuahuan Desert in New Mexico.

##### Additional specimens examined.

U.S.A. • California, Riverside Co., Sonoran Desert, Joshua Tree National Park, along seasonal stream bed, 33.7781, -115.8987 m, alt. 1005, on gneiss, 9 Nov. 2012, K. Knudsen 15197 (SBBG); • San Bernardino Co., Mojave Desert, Superior Valley, 35.2905, -116.9594, alt. 966 m, on limestone, 20 Apr. 2014, G.R. Ballmar s.n. (hb. K&K); • Nevada, Clark Co., Echo Wash, American Break Road, 36.3918, -114.5294, 349 m, on limestone,

30 Mar. 2009, T. Wheeler 2663 (hb. Wheeler); • Lincoln Co., toe of Chief Range just N of Indian Cove, 37.67, -114.49, alt. 1400 m, top of HCl- tuff boulder, 8 May 2016, J. Hollinger 11450 (BRY-C); • Nye Co., Golden Gate Range, 38.186, -115.347, alt. 1760 m, on rhyolitic HCl- tuff, 10 May 2016, J. Hollinger 11125, & N. Noell (BRY-C); • New Mexico, Lincoln Co., Tularosa Basin, Chihuahuan Desert, Oscura, 33.485, -106.0930, alt. 1590 m, on HCl- sandstone, 17 Mar. 2022, K. Knudsen 19434 & J. Kocourková (hb. K&K); • 33.7696, -106.0928, on HCl- sandstone, 14 Mar. 2022, J. Kocourková 10850, 10687 (hb. K&K); • Carrizozo Malpais, Valley of Fire Recreational Area, slope below lookout, 33.6788, -105.59, alt. 1579 m, on HCl- sandstone, 19 Mar. 2020 K. Knudsen 19373.1 & J. Kocourková (hb. K&K); • on slope above lava beds, 33.6816, -105.9283, alt. 1590 m, on HCl- sandstone, 21 Mar. 2020, K. Knudsen 19408, 19413 & J. Kocourková (hb. K&K); • Kane Co., on sandstone ledges, just north of Hog Canyon, north Kanab and east of US Highway 89. 37.089, -112.537, alt. 1650 m, on Navajo sandstone escarpments, 10 May 2024, S. Leavitt 24038 (BRY-C); • Uintah Co., Dinosaur National Monument, 40.26620, -109.15822, no elevation, on HCl- red sandstone, 27 Sept. 1991, L. St. Clair 424 (BRY-C).

##### Notes.

*Acarospora coloradiana* differs from *A. peltata* in having small globose ascospores in maturing asci, like *A. lactea*, and mature ascospores often 2–3 × 2–4 μm, globose to subglobose, oftena few short and thin ellipsoid, while *A. peltata* has wide range of ellipsoid ascospore sizes. *Acarospora coloradiana* also differs in usually having euamyloid hymenial gel which is rarely unstable, but never completely hemiamyloid, while *A. peltata* is always hemiamyloid. It is possible, not using our protocol, to usually get a euamyloid reaction, and Magnusson recognized it having a euamyloid hymenial gel without a protocol (Magnusson 1929). *Acarospora coloradiana* differs from *A. lactea* in having a usually translucent vs. non-translucent surface when wet.

#### Acarospora lactea

Taxon classificationFungiAcarosporalesAcarosporaceae

K. Knudsen, Kocourk., S. D. Leav. & T. B. Wheeler sp. nov.

20FB2719-7491-5C9E-B580-548182A312BF

863900

Figs 7, 8, 9

##### Type.

U.S.A. Montana, Choteau Co., Missouri River Breaks, Carter ferry, Missouri River Breaks, 853 m, on hard sandstone, 47.7523, -110.9065, alt. 1176 m, 2 May 2011, T. Wheeler 3395 (ASU, holotype).

**Figure 8.**
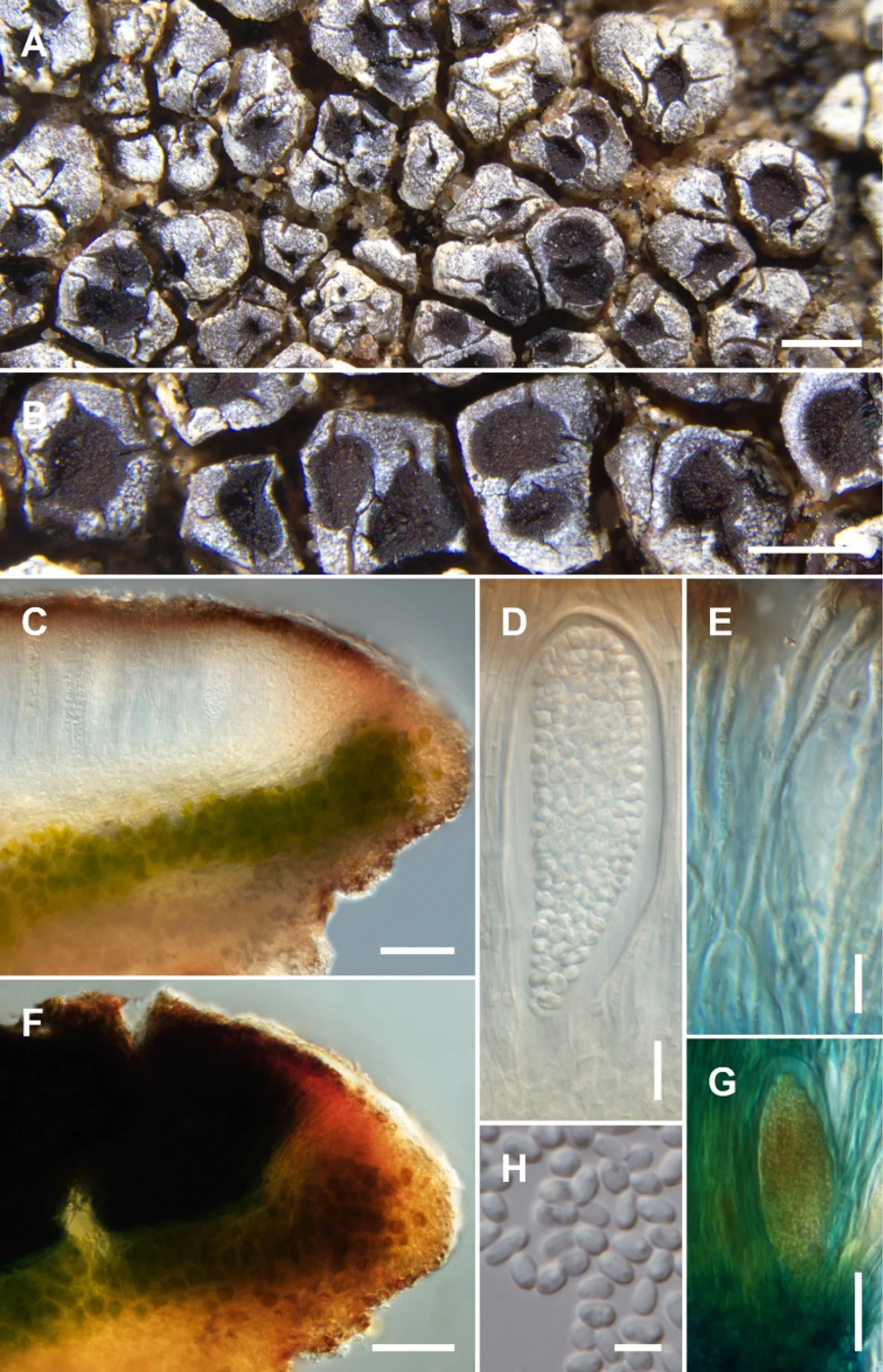
*Acarospora lactea*, Kocourková 11819. **A**. Thallus of convex, broadly attached areoles with apothecia; **B**. Detail of areoles filled with one to two dark reddish brown apothecia; **C**. Vertical section through the apothecium showing reddish brown epithecium, thin parathecium and continuous algal layer below apothecium; **D**. Ascus with young globose to subglobose ascospores; **E**. Paraphyses anastomosed in the lower part of hymenium with slightly expanded ends in a thin gel cap; **F**. Hymenial gel IKI+ dark blue; **G**. Ascus wall, hymenial gel and subhymenium showing amyloid reaction in IKI; **H**. Broadly ellipsoid ascospores released from asci. Scale bars: 1 mm (**A**); 500 µm (**B**); 50 µm (**C, F**); 20 µm (**G**); 10 µm (**D, E**); 5 µm (**H**).

**Figure 9.**
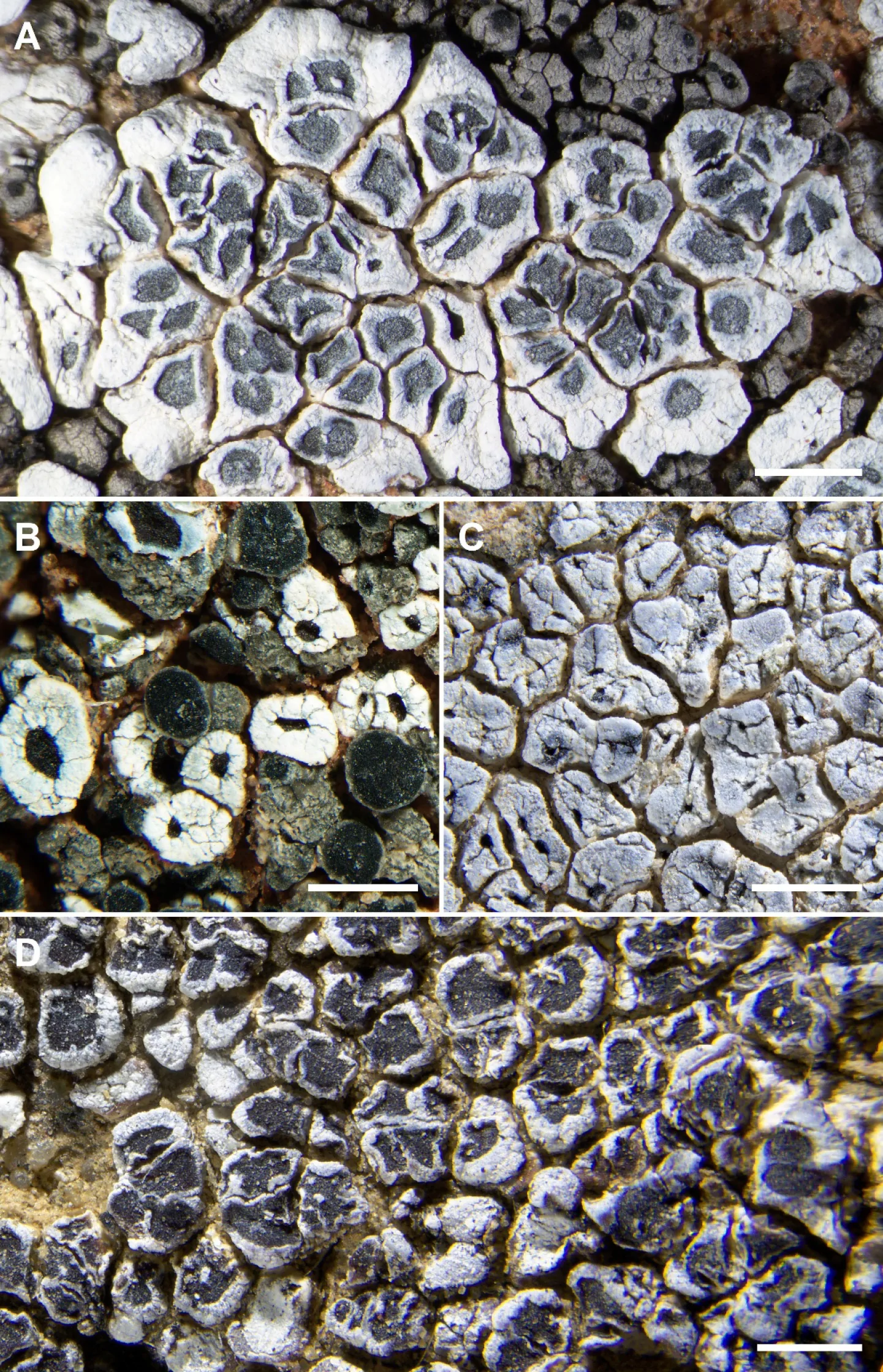
*Acarospora lactea*. Wheeler 2928 (**A**), Hollinger 17495 (**B**), Hollinger 10975 (**C**), Wheeler 3395 – Holotype (**D**). **A–D**. Variability of the thallus; **B**. Thallus parasitic on *Pyrenodesmia cf. albovariegata*. Scale bars: 1 mm.

##### Diagnosis.

Similar to *Acarospora coloradiana* in having euamyloid hymenial gel, but differing in having a non-translucent pruinose epicortex vs. translucent when wetted and brown or gray.

##### Etymology.

Named for the beautiful non-translucent milkywhite epicortex of most areoles or squamules when wet.

##### Description.

Hypothallus endosubstratal, no algae observed. Thallus of scattered or contiguous areoles or squamules, 0.4–1.5 mm wide, 200–300(–600) mm thick, usually broadly attached or with mycelial base or narrowing to a stipe. Upper surface white, not translucent in water, with or without fissures, replicating by division. Lower surface white, narrow and ecorticate. Epicortex of pruina in a well-developed polysaccharide matrix, 10–60 μm thick. Eucortex thin, 25–40 µm tall, of hyphae up to 3 μm wide, breaking up into angular or round autonomous units, upper layer reddish-brown, lower layer hyaline. Algal layer 90–200 μm thick, rarely thicker, uninterrupted by hyphal bundles, algae 8–10 μm wide. Medulla 200–300 μm, rarely thicker, of thin-walled hyphae up to 4 μm wide, branching, obscure with substrate crystals. Apothecia usually 1, rarely 2–4, up to 1.7 mm wide, immersed, sometimes apothecia reducing the areole or squamule to a thalline margin, rarely elevated in a thalline margin, disc black, usually not pruinose, usually reddish-brown when wetted. Parathecium indistinct to 40–50 μm wide, hyphae 1–2 μm, hard to distinguish from hymenium, IKI-, sometimes merging with cortex, sometimes forming a narrow ring around apothecia. Hymenium 100–130(–150) μm tall, epihymenium 10–15 μm tall, reddish-brown, paraphyses 1–2 μm wide, apices barely expanded to 3 μm wide, usually in gel caps 2–5 μm wide, sometimes with black pigment mark, hymenial gel IKI+ dark blue. Asci cylindrical to clavate, 60–90(–120) × (10–)20–40 μm, ascospores 3.5–6.0 × (2.0–)2.5–3.5 μm, ellipsoid to broadly ellipsoid. Subhymenium interspersed with oil drops in Montana specimens, but not seen in other specimens, 20–50 μm tall, or V-shaped up to 100 μm tall, of intricate hyphae with some swollen cells, IKI+ dark blue, euamyloid. Hypothecium 100–150 μm tall, hyphae 2 μm wide or surrounding a V-shaped subhymenium, IKI-. Pycnidia globose up to 200 μm wide, conidiogenous cells ca. 15 × 1 μm, conidia 2 × 1 μm. Not producing secondary metabolites.

##### Habitat and distribution.

United States (Montana, Nevada, Utah). Best developed along Missouri River Breaks in Montana, often smaller in deserts in Nevada and Utah. Usually on calcareous rock, sometimes HCl- rock. In several specimens, the holotype, Hollinger 17495, 10972 and Wheeler 2928, *Acarospora lactea* is parasitic, overgrowing other lichens in competition for space, but with no sign of morphing out of host’s thallus. Reports from Colorado and other adjoining states as well as Mexico are expected because it is a newly-described species.

##### Additional specimens examined.

U.S.A. • Montana, Choteau Co., Missouri River Breaks, Hole in the Wall, 47.7994, -110.0450, alt. 853 m, on HCl+ sandstone,13 Oct. 2009, T. Wheeler 2928 (PRM); • Carter ferry, Missouri River Breaks, on hard sandstone, 47.7523, -110.9065, alt. 853 m, 2 May 2011, T. Wheeler 3392 (topotype, ASU); • Hill Co., Fresno Reservoir, 48.6863, -110.0207, alt. 808 m, on HCl+ sandstone, 26 Oct. 2010, T. Wheeler 3624 (ASU); • Nevada, Lincoln Co., foot of escarpment, rock on limestone, 37.751, -114.336, alt. 1550 m, 1 Apr. 2016, J. Hollinger 10975 (BRY-C); • Big Hogback, 37.447, -114.334, alt. 1600 m, on limestone rock on ground, 1 Apr. 2016, J. Hollinger 10972 (BRY-C); • Utah, Grand Co., Tushar Canyon, Book Cliffs, 39.0992, -110.334, alt. 1402 m, on HCl- sandstone, 4 Apr. 2011, T. Wheeler 3255, 3256 (ASU); • vicinity of Fisher Towers, north of Moab, 38.7224, -109.3058, alt. 1550 m, on HCl+ red sandstone, 27 Jan. 2018, S. Leavitt 18011 (BRY-C); • Iron Co. 39.107, south of Parawon, Dixie National Forest, along FR #049 in First Left Hand Canyon, vicinity of Noah’s Ark (Noah’s Ark Trail #32005), 29.107, -110.669, alt. 1600 m, on HCl+ sandstone, 1 Jun. 2024, S. Leavitt 24240, 24242 (BRY-C); • San Juan Co., Salt Creek, narrow part of mesa ca. 2 km WSW of Salt Creek Trailhead beside Beef Basin Rd., 37.9399, -109.7260, alt. 2204 m, steep N slope just below rim of sandstone mesa with pinyon-juniper woodland, parasitic on Pyrenodesmia cf. albovariegata on sandstone, 15 May 2017, J. Hollinger 17495 (hb. K&K); • canyon of Indian River on regional road SR211 between the Canyonlands National Park and Hwy US 191, 38.0234, -109.5397, alt. 1740 m, on large boulder of consolidated calcareous sandstone, 30 Sept. 2025, J. Kocourková 11819 & K. Knudsen (hb. K&K).

#### Acarospora nevadensis

Taxon classificationFungiAcarosporalesAcarosporaceae

H. Magn., Kungl.-Akad. Handlingar 7, 4: 322 (1929)

C3279BC0-F2EB-510E-8EC8-0B2945B461CE

375640

Fig. 10

##### Type.

U.S.A. • Nevada: Reno, on rock, alt. 1524 m, Dec 1909. A. C. Herre (UPS! holotype, collector misidentified as H. E. Hasse), isotype (NY!).

**Figure 10.**
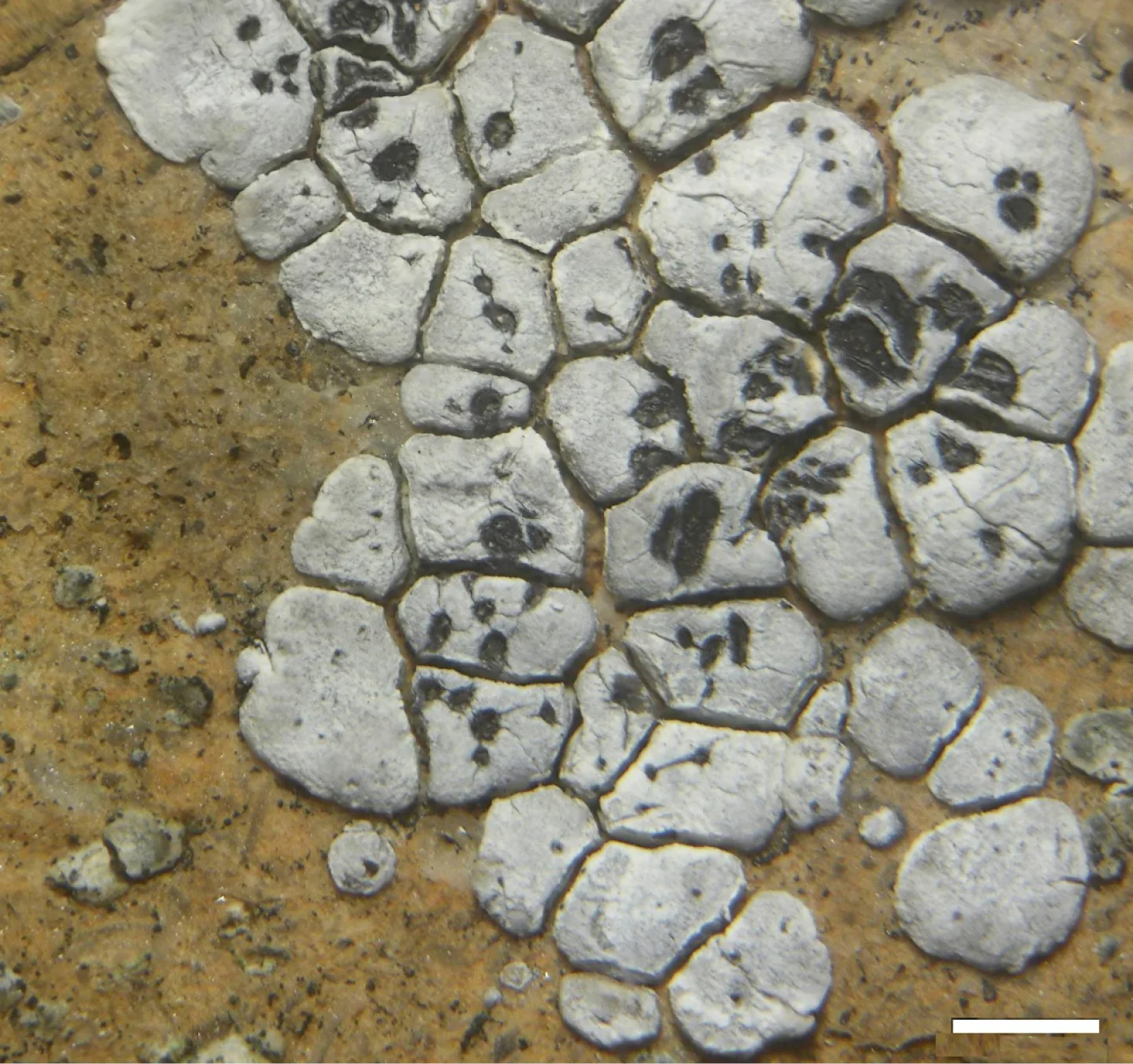
*Acarospora nevadensis*, Hollinger 16963. Habit of the thallus with apothecia. Photograph by Jason Hollinger. Scale bar: 1 mm.

##### Description.

Hypothallus endosubstratal, no algae observed. Thallus of areoles or squamules, rarely more than 2–3 cm wide, areoles irregular, 0.4–1.5 mm in diam., up to 0.9 mm thick, usually contiguous, flat to convex, replicating by division. Upper surface densely white pruinose to dark reddish-brown and epruinose, smooth or fissured to deeply dissected. Epicortex 10–30 µm thick, thin in epruinose specimens. Eucortex 40–60 µm thick, cells globose 1.5–4.0 um wide, upper layer yellowish to reddish-brown, 20–30 µm thick, lower layer hyaline. Algal layer 100–150 µm tall, algal cells 8–12 µm wide, uninterrupted, thin to absent below apothecia, pushed to sides of the areoles with the expansion of the apothecium. Medulla 200–400 µm thick, clear or obscured by substrate crystals, hyphae intricate, 2–5 µm wide. Apothecia usually one per areole or squamule, immersed, disc dark brown to black, smooth or rough, pruinose or not. Parathecium indistinct to 20 µm thick, IKI-. Hymenium 60–120 µm tall, epihymenium reddish-brown, 10–15 µm tall, paraphyses 1.5–2.0 µm wide at base, apices expanded to 2.0–2.5 µm or not, moderately branched, hymenial gel IKI+ red, weak or strong or blue turning red, hemiamyloid. Asci clavate, 65–80 × 20–28 µm, ascospores short and broadly ellipsoid, 3–5 × 2.5–3.0 µm. Subhymenium hyaline, 20–35 µm thick, IKI+ blue, euamyloid. Hypothecium up to 20 µm thick, IKI-. Pycnidia: not observed. Secondary metabolites: gyrophoric acid, cortex and/or medulla, C+ red, KC+ red, reaction strong to uneven.

##### Habitat and distribution.

Mexico, Baja California. U.S.A. (Arizona, California, Idaho, New Mexico, Nevada, Utah), on calcareous and non-calcareous rock.

##### Selected specimens examined.

Mexico • Baja California, 16 km [10 miles] S of Santa Ynez, 29.6666, -114.7, no elevation, on volcanic rock, 25 May 1974, T. H. Nash III 8830 (ASU); • U.S.A., Arizona, Coconino Co., edge of Gray Mesa, 31 km [19 mi.] SE of Page, 36.7, -111.1833, alt. 1800 m, on sandstone, 22 May 1973, T. H. Nash III 6490 (ASU); • Leche Rock, 9.7 km [6 miles] E of Page, 36.8666, -111.3166, alt. 1620 m, epruinose on sandstone, 21 Sep 1970, T.H. Nash III 5053 (ASU); • Yavapai Co., 1 km N of McGuireville, 34.6416, -111.8166, alt. 1070 m, on limestone, 23 Feb 1999, T.H. Nash III 5053 (ASU); • Graham Co., Coronado National Forest, Pinaleno Mountains, route 366, Mt. Graham, 3.2 km above Coronado National Forest boundary, 32.7166, -109.7333, alt. 1220 m, on rock, July 4, 1972, T. H. Nash III 4182 (ASU); • Yuma Co., Kofa Wildlife Refuge, dirt road to “Horse Tanks”, 933.25, -114.2333, alt. 610 m, on calcareous rock, Jan 8 1986, Bruce Ryan 16027 (ASU); •Granite Mountain (Bighorn Basin), shaded N-facing seasonal stream, epruinose probably from flushing, 34.7847, -115.6725, alt.1588 m, 2 Dec 2005, K. Knudsen 4386 & Silke Werth (SBBG); • Idaho, Owyhee, Mudflat Road, just north of small cemetery, south of Grandview, T7S R3E S4, 42.85, -116.1, alt. 930 m, on limestone cliffs, Jun 19991, Bruce McCune 19081a (OSU); • Nevada, Lincoln County, Mormon Mountains, ca. 7 km NNE of Mormon Peak, 37.02, -114.462, alt. 1550 m, 24 Apr 2024, Jason Hollinger 16963 & N. Noell (BRY-C); • Pershing Co., Kamma Range, Rosebud Canyon, ca. 3 km ENE of Barrell Springs., 40.7911, -118.6711, alt. 1460 m, on rhyolite, J. Hollinger 22300, 15 Sep 2018 (NY, hb. Hollinger); • New Mexico, San Juan, Hogback North, a large dirt mound approximately 32 km [20 mi] N of Fourming, on right side of Hwy 550, no elevation, on rock, 19 Sep 1973, Yvonne Rogers 28 B (ASU); • Utah, San Juan County, Bridger Jack Mesa, base of cliff at top of gully ca. 400 m, S of small camp on bench on N side of road ca. 14 km south on Beef Basin Road from park road, 37.9815, -109.624, alt. 2000 m, on calcareous sandstone, 5 May 2017, J. Hollinger 17423 (hb. H&N).

##### Notes.

The epruinose specimens have not been sequenced, but have similar brown areoles and broadly ellipsoid ascospores. They could represent another species. They are rare too and usually collected around some lightly pruinose specimens, often in areas flushed by water. Both produce gyrophoric acid in the cortex and sometimes also into the medulla.

The Chinese *Acarospora tianshanica* was described as producing gyrophoric acid (Nurtai et al. 2017). Recently, it was found that part of the sequenced specimens do not produce any secondary metabolites (Liang et al. 2026).

#### Acarospora peltastica

Taxon classificationFungiAcarosporalesAcarosporaceae

Zahlbr., Beihefte zum Botanischen Centralblatt 13: 161 (1902)

2F371EF6-8D92-5F7D-B984-7C1F9CCB0740

375665

##### Type.

U.S.A. • California, Riverside Co., Palm Springs, Sonoran Desert, on granite, lacking date, H. E. Hasse 816 (W, n.v., presumed lost).

##### Description.

Thallus dispersed or contiguous, areoles 2.0–4.0 mm wide, 0.5–0.8 mm thick, broadly attached, replicating by division. Upper surface white pruinose, sometimes with bluish tint. Epicortex (50 –)110–130 µm thick, with deep fissures V-shape, cleaving through epicortex to eucortex. Eucortex 40–50 µm thick, upper layer reddish-brown, lower layer hyaline, cells round, 2–3 µm wide. Algal layer 100–120 μm thick, uninterrupted, algal cells 9–18 μm wide. Medulla 300–500 μm thick, upper layer clear, lower layer obscure with substrate crystals, hyphae 4–5 μm wide, thin-walled, loosely intricate to anticlinal at base. Apothecia 1–3, immersed, 0.5 mm wide, disc dark. Parathecium 15 μm wide, IKI+ pale blue according to Magnusson (1929), but doubtful, probably stained by initial hymenial reaction. Hymenium 100–110(120–130) μm tall, paraphyses 2 μm wide, hymenial gel IKI+ light blue or turning a dirty dark vinous red, hemiamyloid. Asci 70–80 × 15–17 μm, ascospores globose (3.5–)4–5 μm. Subhymenium, 50–70 μm tall, IKI+ pale blue. Hypothecium 30–35 μm tall, no IKI data. Pycnidia not observed. No secondary metabolites reported. This description is based on Zahlbruckner’s description of holotype (1902) and Magnusson (1929).

##### Distribution and ecology.

California, Riverside County, Palm Springs (4 specimens); Los Angeles Co., Santa Monia Mountains (1 specimen) (Zahlbruckner 1902; Magnusson 1929), all collections by H. E. Hasse. These would have been collected on non-calcareous rock. *Acarospora peltastica* is a rare species in California and is sympatric with *A. peltata* and *A. coloradiana* in southern California.

##### Notes.

*Acarospora peltastica* was collected by Hasse and described from Palm Springs, California by Zahlbruckner. It is only known from the lost holotype (Zahlbruckner 1902) and four collections reported by Magnusson (Magnusson 1929). It has not been reported again in California. The holotype is presumed lost. The authors have never seen a specimen. It would look like a robust specimen of *A. peltata* (Magnusson 1929). It is possible specimens have been overlooked by collectors not sectioning the apothecia because all specimens with the *strigata* phenotype in North America were determined as *A. strigata* since Magnusson (1929). Recently, the first author (K.K.) used *A. peltastica* and *A. strigata* group in a key to designate members of *A. strigata* group in North America with hemiamyloid hymenial gel, but without a description and before the completion of our current study of the *strigata* group (Knudsen et al 2023a). The description of *A. peltastica* for specimens with ellipsoid ascospores is based on Hasse (1913) who stated *A. peltastica* had globose ascospores but also long ellipsoid ascospores, lumping *A. peltata* with *A. peltastica*. Hasse often determined specimens with ellipsoid ascospores as *A. peltastica*. Zahlbruckner's Lichenes Rariores Exsiccati 75, collected by Hasse, had one specimen at least of *A. peltastica* s. str. with globose ascospores according to Magnusson (1929). All of the collections we examined had ellipsoid ascospores in Zahlbruckner's Lichenes Rariores Exsiccati 75 and were *A. peltata* (Knudsen and Lendemer 2005). *Acarospora coloradiana* also occurs in southern California and could also be mixed in Zahlbruckner's Lichenes Rariores Exsiccati 75.

#### Acarospora utahensis

Taxon classificationFungiAcarosporalesAcarosporaceae

H. Magn., Magnusson, Acta Horti Gotoburgensis 19: 31–49 (1952) (Holotype, UPS!)

CA63718F-A197-5A7C-8365-392FF4A91DD8

3639169

Fig. 11

##### Type.

U.S.A. • Utah, Wayne, Ekker’s Ranch, alt. 1830 m, on dry sandstone, 19 May 1950, S. Flowers (UPS! holotype).

**Figure 11.**
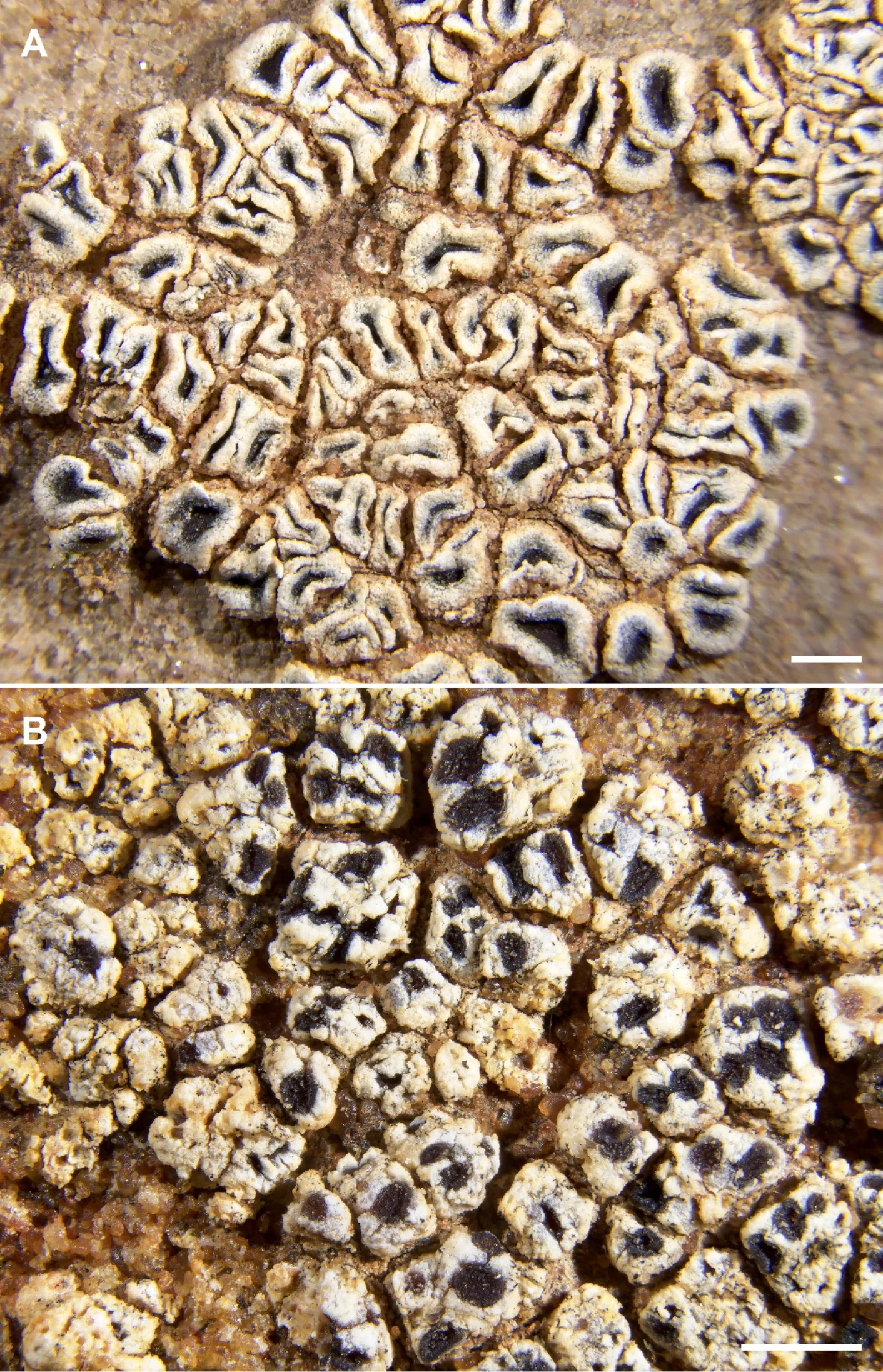
*Acarospora utahensis* Hollinger 7332 (**A**), Hollinger 6316 (**B**). **A**. Habit of the thallus, apothecia fully filling areoles; **B**. Areolate thallus with apothecia. Scale bars: 1 mm.

##### Description.

Hypothallus in substrate, no algae observed. Thallus an areolate crust, covering areas up to 10 cm, replicating by division, areoles 0.2–2.0 mm wide, up to 600 µm tall, broadly attached to substrate. It has a flat topography until areoles become mature and swell, varying in height. Upper surface with or without fissures, whitish when dry, gray-brown when wet. Epicortex 20–60 µm thick. Eucortex 40–60 µm thick, irregular cells to 5 µm wide, upper layer red brown, lower layer hyaline. Algal layer 70–100 µm thick, continuous or absent under apothecium, cells mostly 10 µm wide. Medulla 200–300 µm thick, gray, filled with substrate crystals, hyphae obscured, intricate, 2–5 µm wide. Apothecia 0.1–0.4 mm wide, black looking, usually one per mature areole. Disc usually black when dry or wet, rough, immersed. Parathecium expanding around apothecium up to 35 µm, merging with cortex, sometimes forming a raised thalline margin, IKI-. Hymenium (100–)120–170(–200) µm tall, epihymenium reddish-brown, ca. 20–30 µm tall, paraphyses 1–2 µm wide, apices unexpanded in gel caps with black pigment marks, epihymenium IKI+ dark brownish-red, while the hymenium is IKI+ dark blue, but especially if squashed, IKI reaction of the epihymenium spreads and turns parts or all of the hymenium dark brownish-red. Asci 90–100 × 18–21 µm, ascospores 9–13 × 4–5 µm. Subhymenium 30–50 µm tall, indistinct from base of hymenium, IKI+ blue. Hypothecium ca. 20 µm tall, distinct from medulla, IKI-. Pycnidia common on young areoles, a black dot, 180–200 × 60 µm (variable width), conidiogenous cells ca. 15 × 1 µm, conidia mostly 1 × 0.5 µm. Not producing secondary metabolites.

##### Specimens examined.

USA • Utah, Wayne Co., ca. 32 miles west of entrance to Canyonlands National Park, 38.2438, -110.2152, alt. 1866 m, on loosely consolidated HCl- sandstone, 11 Apr 2014, J. Hollinger 7327, 7332 & N. Noell (hb. H&N, hb. K&K).

##### Notes.

We, like Magnusson (1952), have used an IKI+ euamyloid hymenial gel as a key character for *A. utahensis*, but the epihymenium is IKI+ reddish and ultimately turns whole hymenium slowly reddish-brown, hemiamyloid (Knudsen et al. 2023a). We base determinations on ascospore size.

### Key to *Acarospora strigata* group in North America

If you are not sure you have a *strigata* specimen, look at all the pictures. Or use our key in Knudsen et al. (2023a) to determine the *strigata* group, but use this key for final determination. The most common species is *A. peltata*. The rarest species are *A. peltastica* (California) and *A. utahensis* (Utah). *Acarospora coloradiana* and *A. lactea* are not as frequent as *A. peltata* and apparently have more limited distributions.

The key works well if you have relatively fresh Lugol’s of most formulas. We use a protocol for repeatable results. We use only Merck’s Lugol for the gram-staining method, Sigma-Aldrich 1.09261. It is kept in dark glass bottles in the dark, except when in use and the IKI is replaced every month. The bottle is shaken before each test. A thin section of the lichen is placed under a coverslip in a drop of water and IKI is added to test the first reaction. Then the section is squashed to allow full saturation for second reaction. The reactions are either euamyloid (dark blue) or hemiamyloid (red). In squash preparations, sometimes blue reactions turn red and are recorded as hemiamyloid. Pale blue or blue with greenish or other tints, usually means your IKI is old. If the subhymenium is not IKI+ blue, but if red, you definitely have too much I in the formula you are using.

One should at least be familiar with the whole paper to be aware of secondary characters or problems which may be rare, but will help determine specimens. Examples are where a wet cortex does not turn brown in *A. peltata* and *A. coloradiana* and discussion of hymenial gel stains for *A. coloradiana* and *A. utahensis*.

**Table:** 

1	Producing gyrophoric acid (C+/KC+ cortex)	***Acarospora nevadensis***
–	Not producing gyrophoric acid	**2**
2	Ascospores broadly ellipsoid, always 9–13 × 4–5 µm	***Acarospora utahensis***
2	Ascospores not always broadly ellipsoid, not always 9–13 × 4–5 µm	**3**
3	IKI+ hemiamyloid hymenial gel	**4**
–	IKI+ euamyloid hymenial gel	**5**
4	Ascospores ellipsoid to broadly ellipsoid, (2.0–)4–6(–10) × 1–3 μm, the most common species in North America	***Acarospora peltata***
–	Ascospores globose (3.5–)4–5 μm, the rarest species	***Acarospora peltastica***
5	Usually translucent white epicortex when wet, usually turning brown or gray	***Acarospora coloradiana***
–	Non-translucent white epicortex when wet, usually turning brighter white	***Acarospora lactea***

## Supplementary Material

XML Treatment for Acarospora strigata

XML Treatment for Acarospora peltata

XML Treatment for Acarospora coloradiana

XML Treatment for Acarospora lactea

XML Treatment for Acarospora nevadensis

XML Treatment for Acarospora peltastica

XML Treatment for Acarospora utahensis

## Additional information

### Artificial Intelligence (AI) use

The authors accept full responsibility for the content of the manuscript, including the disclosure of any use of AI.

No AI tools were used in the preparation of this manuscript.

### Author ORCIDs

K. Knudsen https://orcid.org/0000-0001-5419-5729

J. Kocourková https://orcid.org/0000-0001-5511-9752

E. Kondrysová https://orcid.org/0000-0002-8337-3253

S. Leavitt https://orcid.org/0000-0002-5034-9724

J. Hollinger https://orcid.org/0000-0003-2465-2487

N. Noell https://orcid.org/0009-0005-7563-6921

J. Malíček https://orcid.org/0000-0002-3119-8967

T. Wheeler https://orcid.org/0000-0003-0668-8662

### Data availability

All of the data that support the findings of this study are available in the main text or Supplementary Information.

## Supplementary materials

Supplementary material 1 Sampled specimens included in the molecular phylogeny Data type PDF https://binary.pensoft.net/file/1813439This dataset is made available under the Open Database License (http://opendatacommons.org/licenses/odbl/1.0/). The Open Database License (ODbL) is a license agreement intended to allow users to freely share, modify, and use this Dataset while maintaining this same freedom for others, provided that the original source and author(s) are credited. Kerry Knudsen, Jana Kocourková, Eva Kondrysová, Steve Leavitt, Jason Hollinger, Natassja Noell, Jiří Malíček, Tim Wheeler

Supplementary material 2 Maximum Likelihood tree obtained by phylogenetic analysis using a combined data set of ITS, mtSSU and nLSU sequences of 60 members of *Acarosporaceae* Data type PDF Explanation note Maximum Likelihood bootstrap values (ML ≥ 0.70) are indicated above branches. Pycnora sorophora was used as an outgroup. https://binary.pensoft.net/file/1813440This dataset is made available under the Open Database License (http://opendatacommons.org/licenses/odbl/1.0/). The Open Database License (ODbL) is a license agreement intended to allow users to freely share, modify, and use this Dataset while maintaining this same freedom for others, provided that the original source and author(s) are credited. Kerry Knudsen, Jana Kocourková, Eva Kondrysová, Steve Leavitt, Jason Hollinger, Natassja Noell, Jiří Malíček, Tim Wheeler
