# Supplementary material for: White and fissured: the Acarospora strigata group (Acarosporales, Acarosporaceae) in North America
Source: MycoKeys. 2026 Oct 19;142:73–102. doi: 10.3897/mycokeys.142.208759 (PMC13717517; doi:10.3897/mycokeys.142.208759)
Supplement: Supplementary file 1 [file mycokeys-142-073-s001.pdf]

Supplemental Materials No 1. Table of sampled specimens included in the molecular phylogeny. Specimens collected by Kocourková and Kundsen (Hb. K&K), Hollinger & Noell (hb. H&N), Jiří Malíček, and Tim Wheeler are in their private herbaria unless otherwise stated.

| Species | Origin | Voucher | ITS | mtSSU | nLSU |
|---|---|---|---|---|---|
| *Acarospora agostiniana* | U.S.A., New Mexico, Organ Mountains | Knudsen 19236 (SBBG) | ON707085 | ON715682 | ON725172 |
| *Acarospora amabilis* | U.S.A., New Mexico, Chihuahuan Desert, Dona Ana Co. | Knudsen 19086.2 (SBBG) | ON707092 | ON715687 | ON725177 |
| *Acarospora americana* | U.S.A., Utah, Millard Co., Pavant Mts., Fillmore | Kocourkova 11607.2 | PZ456483 | PZ456480 | PZ456487 |
| *Acarospora applanata* | U.S.A., New Mexico | Knudsen 19245 | ON447631 | ON367904 | ON391459 |
| *Acarospora badiofusca* | Austria, Steiermark, Schladminger Tauern | Turk 32318 (GZU) | OP162390 | OP177774 | OP216707 |
| *Acarospora boulderensis* | U.S.A., Kansas, Wilson Lake | Morse 15457a | OP162359 | OP177746 | OP216677 |
| *Acarospora carnegiei* | U.S.A., New Mexico, Organ Mountains | Knudsen 19259 (SBBG) | MW989383 | MW989432 | MW989402 |
| *Acarospora carrizoensis* | U.S.A., San Luis Obispo Co. | Dart 1426 (SBBG) | OQ171053 | OQ184769 | OQ195823 |
| *Acarospora cervina* | Czech Republic, Central Bohemia, Loděnice | Kocourkova 10492 | ON794168 | ON787648 | - |
| ***Acarospora coloradiana*** | U.S.A., New Mexico, Chihuahuan Desert | Knudsen 19408 | ON794179 | ON787661 | ON964954 |
| ***Acarospora coloradiana*** | U.S.A., New Mexico, Chihuahuan Desert | Knudsen 19373 | ON794180 | ON787662 | ON964955 |
| ***Acarospora coloradiana*** | U.S.A., New Mexico, Lincoln Co. Oscura | Knudsen 19434 | OQ171060 | OQ184776 | OQ195830 |
| ***Acarospora coloradiana*** | U.S.A., Nevada, Clark Co. (hb. Wheeler) | Wheeler 2663 | PZ456484 | OP902964 | - |
| *Acarospora contigua* | U.S.A., New Mexico | Knudsen 19148 (SBBG) | MW989384 | MW989433 | - |
| *Acarospora divisa* | U.S.A., New Mexico, Chihuahuan Desert | Knudsen 19384 (ASU) | OK142748 | OK032133 | PZ456488 |
| ***Acarospora epilutescens*** | Mexico, Nuevo Leon | Huereca UN-455 (hb. K&K) | OK142741 | OK032126 | PZ456489 |
| *Acarospora fissurata* | U.S.A., New Mexico, Dona Ana Co., Chih. M., Organ M., Las Cruces | Kocourkova 10255 (PRM) | OK142742 | OK032127 | PZ456490 |
| *Acarospora fuscata* | Norway | Tonsberg 45807 | ON447607 | ON367880 | ON391440 |

| | | | | | |
|---|---|---|---|---|---|
| *Acarospora heufleriana* | U.S.A., Utah, Sevier Co. (hb. Wheeler) | Wheeler 3287 | OP887977 | OP902957 | - |
| *Acarospora chrysops* | U.S.A, New Mexico | Knudsen 19385 | ON447623 | ON367896 | ON391451 |
| *Acarospora imbriculata* | Italy, Valle d´obosta | Buschardt 2187 (B) | OP162381 | OP177765 | - |
| *Acarospora indistincta* | U.S.A., Mojave Desert, Joshua Tree NP | Knudsen 5782 (SBBG) | ON707096 | ON715691 | ON725180 |
| *Acarospora irregularis* | Czech Republic, Central Bohemia, Prague, Motol | Malicek 2128 | OP162363 | OP177750 | OP216681 |
| *Acarospora joshuaensis* | U.S.A., California, Mojave Desert | Knudsen 13112.3 (SBBG) | OP162387 | OP177771 | OP216704 |
| ***Acarospora lactea*** | U.S.A., Utah, Grand Co. (hb.Wheeler) | Wheeler 3256 | OP887973 | OP902953 | PZ456491 |
| ***Acarospora lactea*** | U.S.A., Missouri River breaks | Wheeler 3395 (ASU) | ON707075 | ON715671 | - |
| ***Acarospora lactea*** | U.S.A., Montana (hb. Wheeler) | Wheeler 3624 | OP887968 | OP902946 | - |
| ***Acarospora lactea*** | U.S.A., Montana | Wheeler 3398 | ON447580 | ON367853 | OP962149 |
| ***Acarospora mayrhoferi*** | Italy, Trentino-Alto Adige | Kocourkova 9007 | MZ262719 | MZ262731 | MZ262742 |
| ***Acarospora nashii*** | U.S.A., Nevada, Nye Co. | Hollinger 22699 | PZ456485 | PZ456481 | PZ456492 |
| ***Acarospora peltata*** | U.S.A., Mojave Desert, Joshua Tree NP | Knudsen 14244.3 | ON794175 | ON787657 | PZ456493 |
| ***Acarospora peltata*** | U.S.A., Josua Tree NP, Smoke Tree Wash | Kocourkova 11112 (SBBG) | OR689437 | OR703825 | OR680851 |
| ***Acarospora peltata*** | U.S.A., San Jacinto | Kocourkova 11115 | OR689438 | OR703828 | OR680852 |
| ***Acarospora peltata*** | U.S.A., New Mexico, Eddy Co., Lincoln Natl. Forest | Knudsen 19458 | OQ171058 | OQ184774 | OQ195828 |
| ***Acarospora peltata*** | U.S.A., Utah, Grand Co. (hb.Wheeler) | Wheeler 3232 | OP887971 | OP902950 | OP962158 |
| *Acarospora radicata* | U.S.A., California | Knudsen 13537 | ON447602 | ON367875 | ON391435 |
| *Acarospora sharnoffii* | U.S.A., California, Joshua Tree National Park | Sharnoff 4107 (SBBG) | ON707101 | ON715697 | ON725185 |
| ***Acarospora strigata*** | Chile | Reeb 15-VO2/2 (hb. K&K) | ON447581 | ON367854 | ON391429 |
| *Acarospora tejonensis* | U.S.A., California | Knudsen 18288 (SBBG) | ON447623 | ON367896 | ON391451 |

| | | | | | |
|---|---|---|---|---|---|
| ***Acarospora utahensis*** | U.S.A., Utah, Wayne Co. | Hollinger 7332 | PZ456486 | PZ456482 | PZ456494 |
| *Myriospora hassei* | U.S.A, California, Los Angeles Co. | Knudsen 707 (UCR) | MW715698 | MW715737 | MW715726 |
| *Myriospora hilitzeri* | Czech Republic, Hruby Jesenik, Bělá pod | Malíček 12893 | OP162403 | OP177788 | OP216718 |
| *Myriospora kunyuensis* | China, Shandong | SDNU 20242087 (PRM) | PV176099 | PV176097 | PV176106 |
| *Pleopsidium corrugatulum* | China | CM 20151086 (KUN) | MN129010 | MN129034 | MN129034 |
| *Pleopsidium gobiensis* | China | PC161016-1(KUN) | MN129007 | MN129044 | MN129031 |
| *Pycnora sorophora* | Sweden, Härjedalen | Hermansson 7903a (UPS L-111613) | FJ959357 | AY853338 | AY853387 |
| *Sarcogyne albothallina* | U.S.A., Montana, Chouteasu Co. | Wheeler 3583 (S) | LN810829 | LN810954 | LN810829 |
| *Sarcogyne clavus* | U.S.A., California, Riverside County: Santa Ana Mts. | Kocourkova 10800 | OQ171103 | OQ184815 | OQ195865 |
| *Sarcogyne convexa* | U.S.A., MT, Fergus County, Big snowy Mount. | Wheeler 5743 (PRM) | - | ON787679 | - |
| *Sarcogyne cyclocarpa* | Sweden, Torne lappmark | Westberg P117 (S) | LN810815 | LN810940 | LN810815 |
| *Sarcogyne dispersa* | U.S.A., Colorado | Tripp 10368 (COLO) | PX854503 | PX854516 | PX854529 |
| *Sarcogyne pruinosa* | Czech Republic, Southern Moravia, Netčiny | Malíček 2560 | OQ171097 | OQ184809 | OQ195860 |
| *Sarcogyne pruinosa* | Italy, Abruzzi, Prov. Teramo, S. Stefano | Nimis 24913 (TSB) | OQ171130 | OQ184841 | OQ195890 |
| *Sarcogyne urceolata* | Norway, Soer-Troendelag | Westberg 08-260 (S F123679) | LN810840 | LN810966 | LN810816 |
| *Sarcogyne wheeleri* | U.S.A., MT, Granite Co. | Wheeler 5678 (PRM) | ON794198 | ON787681 | PZ456495 |
| *Timdalia intricata* | Sweden, Härjedalen | Westberg SAR92 (LD) | LN810866 | LN810991 | - |
| *Timdalia intricata* | Sweden, Torne lappmark | Westberg P114 (S) | LN810867 | LN810992 | - |
| *Trimmatothelopsis californica* | U.S.A., California, Monterey County, Cholame Hills | Dart 577 (BRY-C) | OQ171067 | OQ184779 | OP497839 |

| | | | | | |
|---|---|---|---|---|---|
| *Trimmatothelopsis knudsenii* | China, Fujian | Wang 20250041 (PRM) | PV541742 | PV541742 | PV541750 |
| *Trimmatothelopsis mexicana* | Mexico, Presa, Somoretillo, Sabinas Hidalgo | AH-871 (BRY-C) | OK142770 | OK032155 | - |
