# Supplementary material for: White and fissured: the Acarospora strigata group (Acarosporales, Acarosporaceae) in North America
Source: MycoKeys. 2026 Oct 19;142:73–102. doi: 10.3897/mycokeys.142.208759 (PMC13717517; doi:10.3897/mycokeys.142.208759)
Supplement: Supplementary file 2 [file mycokeys-142-073-s002.pdf]

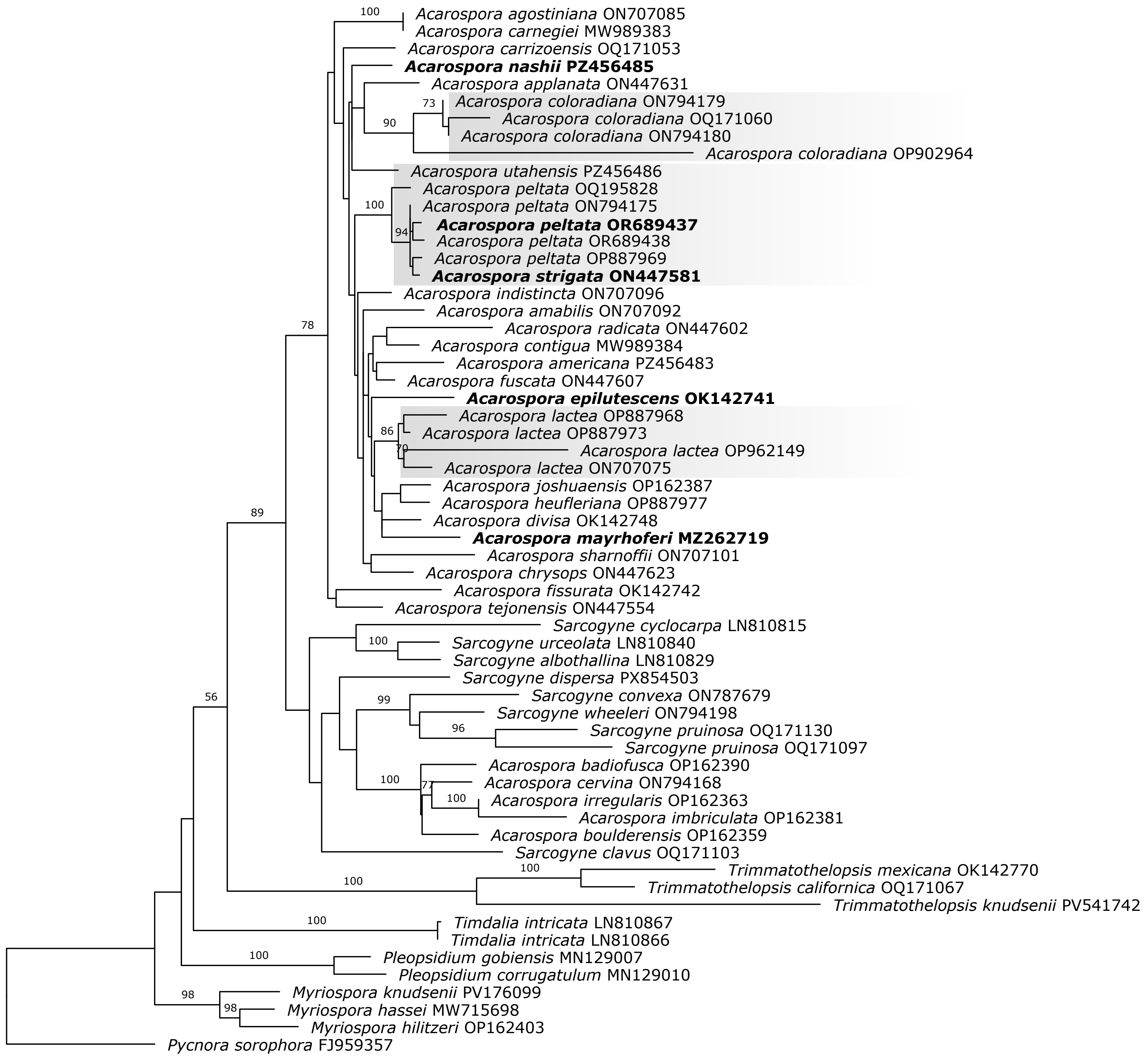

Acarospora agostiniana ON707085
Acarospora carnegiei MW989383
Acarospora carrizoensis OQ171053
Acarospora nashii PZ456485
Acarospora applanata ON447631
Acarospora coloradiana ON794179
Acarospora coloradiana OQ171060
Acarospora coloradiana ON794180
Acarospora coloradiana OP902964
Acarospora utahensis PZ456486
Acarospora peltata OQ195828
Acarospora peltata ON794175
Acarospora peltata OR689437
Acarospora peltata OR689438
Acarospora peltata OP887969
Acarospora strigata ON447581
Acarospora indistincta ON707096
Acarospora amabilis ON707092
Acarospora radicata ON447602
Acarospora contigua MW989384
Acarospora americana PZ456483
Acarospora fuscata ON447607
Acarospora epilutescens OK142741
Acarospora lactea OP887968
Acarospora lactea OP887973
Acarospora lactea OP962149
Acarospora lactea ON707075
Acarospora joshuaensis OP162387
Acarospora heufleriana OP887977
Acarospora divisa OK142748
Acarospora mayrhoferi MZ262719
Acarospora sharnoffii ON707101
Acarospora chrysops ON447623
Acarospora fissurata OK142742
Acarospora tejonensis ON447554
Sarcogyne cyclocarpa LN810815
Sarcogyne urceolata LN810840
Sarcogyne albothallina LN810829
Sarcogyne dispersa PX854503
Sarcogyne convexa ON787679
Sarcogyne wheeleri ON794198
Sarcogyne pruinosa OQ171130
Sarcogyne pruinosa OQ171097
Acarospora badiofusca OP162390
Acarospora cervina ON794168
Acarospora irregularis OP162363
Acarospora imbriculata OP162381
Acarospora boulderensis OP162359
Sarcogyne clavus OQ171103
Trimmatothelopsis mexicana OK142770
Trimmatothelopsis californica OQ171067
Trimmatothelopsis knudsenii PV541742
Timdalia intricata LN810867
Timdalia intricata LN810866
Pleopsidium gobiensis MN129007
Pleopsidium corrugatulum MN129010
Myriospora knudsenii PV176099
Myriospora hassei MW715698
Myriospora hilitzeri OP162403
Pycnora sorophora FJ959357
100
73
90
100
94
78
86
70
89
100
99
96
100
77
100
56
100
100
100
100
98
98
